# Genetic and spatial organization of the unusual chromosomes of the dinoflagellate *Symbiodinium microadriaticum*

**DOI:** 10.1038/s41588-021-00841-y

**Published:** 2021-04-29

**Authors:** Ankita Nand, Ye Zhan, Octavio R. Salazar, Manuel Aranda, Christian R. Voolstra, Job Dekker

**Affiliations:** 1 Program in Systems Biology, Department of Biochemistry and Molecular Pharmacology, University of Massachusetts Medical School, Worcester, MA USA; 2 Biological and Environmental Sciences & Engineering Division (BESE), Red Sea Research Center (RSRC), King Abdullah University of Science and Technology (KAUST), Thuwal, Saudi Arabia; 3 Department of Biology, University of Konstanz, Konstanz, Germany; 4 Howard Hughes Medical Institute, Chevy Chase, MD USA

**Keywords:** Genomics, Epigenomics

## Abstract

Dinoflagellates are main primary producers in the oceans, the cause of algal blooms and endosymbionts of marine invertebrates. Much remains to be understood about their biology, including their peculiar crystalline chromosomes. We assembled 94 chromosome-scale scaffolds of the genome of the coral endosymbiont *Symbiodinium microadriaticum* and analyzed their organization. Genes are enriched towards the ends of chromosomes and are arranged in alternating unidirectional blocks. Some chromosomes are enriched for genes involved in specific biological processes. The chromosomes fold as linear rods and each is composed of a series of structural domains separated by boundaries. Domain boundaries are positioned at sites where transcription of two gene blocks converges and disappear when cells are treated with chemicals that block transcription, indicating correlations between gene orientation, transcription and chromosome folding. The description of the genetic and spatial organization of the *S. microadriaticum* genome provides a foundation for deeper exploration of the extraordinary biology of dinoflagellates and their chromosomes.

## Abstract (web summary)

Genome assembly of the coral endosymbiont *Symbiodinium microadriaticum* shows that genes are arranged in alternating unidirectional blocks and are enriched at the ends of chromosomes. Chromosomes are composed of structural domains separated by boundaries that disappear when transcription is blocked.

## Main

Dinoflagellates are single-celled marine plankton, abundant in the world’s oceans, and of great economic and ecological importance^1^. This is due to their role as primary producers^2^, their ability to cause harmful algal blooms^3^ and because of the symbiotic relationships they form with a broad range of marine invertebrates^1^. In particular, dinoflagellates in the family Symbiodiniaceae^4^ are known for their role as intracellular symbionts of reef-building corals. In recent decades, we have witnessed unprecedented loss of coral reef cover due to local and global anthropogenic insult^5^. Coral bleaching, that is, the loss of Symbiodiniaceae triggered by ocean warming due to climate change, is now the main driver of coral reef degradation^6^.

Dinoflagellates seem to defy many of the cellular features found in other eukaryotes. For instance, dinoflagellates commonly use 5-hydroxymethyluracil instead of thymidine^7^, show a paucity of transcriptional regulation^8–10^, exhibit broad RNA editing^11^ and seem to have a portion of their genes arranged in tandem arrays^12,13^, which may explain, at least partially, the pervasive gene duplication observed in their genomes^14^. Most interestingly, dinoflagellates fold their chromosomes in a way that is distinct from other eukaryotes and that is also distinct from prokaryotes. Dinoflagellates were until recently believed to have no histones, and their DNA was reported to be in a crystal-like state^15,16^. More recent transcriptome studies, however, confirmed that dinoflagellates do possess histones, but lack histone H1 (refs. ^17,18^). However, only a very small fraction of the genome is nucleosomal, as shown, for example, by nuclease digestion patterns^19^. DNA is likely packaged by other proteins, for example histone-like proteins and dinoflagellate/viral nuclear proteins (DVNPs) derived from bacteria and viruses, respectively^19–21^. Dinoflagellate chromosomes appear permanently condensed throughout the cell cycle, and optical birefringence properties of chromosomes suggest they have liquid-crystalline features^22^.

For decades, dinoflagellates have escaped genomic analysis due to their unusually large genomes (ranging from 1 to 250 Gb; ref. ^23^). With the advent of next-generation sequencing, a number of Symbiodiniaceae genome sequences are now available, such as the genomes of *Breviolum minutum*^24^, *Fugacium kawagutii*^25^ and *Symbiodinium microadriaticum*^14^ (among others). These genome sequences are collections of (short) scaffolds, not chromosome-scale assemblies. A chromosome-scale assembly of a dinoflagellate genome is key to providing answers to pertinent biological questions such as how they achieve the unusual folding of their chromosomes, whether the unidirectional alignment of genes is a feature conserved across chromosomes and whether such alignment is related to features of chromosome organization and architecture. Here we generated a chromosome-scale assembly of the genome of the dinoflagellate *S.* *microadriaticum*.

## Results

### Chromosome-scale assembly of the *S.microadriaticum* genome

**Fig. 1:**
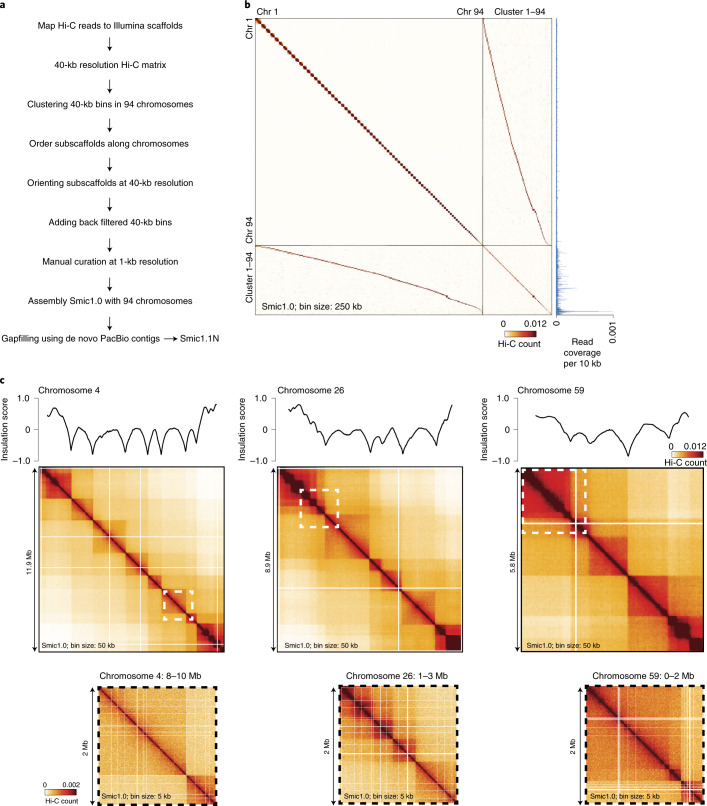
Hi-C-assisted assembly of chromosome-scale scaffolds for *S. microadriaticum*. **a**, Main steps in Hi-C-assisted assembly of Smic1.0 and Smic1.1N. **b**, Hi-C interaction map for the set of 94 chromosomes (ordered by descending size, 250-kb bins) of Smic1.0 and 94 clusters of high-copy scaffolds (ordered according to their preferred interactions with the set of 94 chromosomes). Relative sequence coverage per 10-kb bin is shown along the right axis. Each of the clusters interact mostly with only one of the chromosomes, but sequences in these clusters have, on average, a copy number that is 11 times higher than sequences located along the assembled portions of chromosomes 1–94. **c**, Examples of Hi-C interaction maps for chromosomes 4, 26 and 59. Hi-C data are mapped to Smic1.0 and binned at 50-kb resolution. Dotted squares indicate sections that are shown at higher resolution (5-kb bins) below the chromosome-wide interaction maps. Plots on top of the heatmaps represent insulation profiles (10-kb resolution, window size 500 kb; Methods). This profile represents the number of interactions that occur across each location. Local minima in these profiles indicate the locations of sites across which interaction frequencies are relatively low, and these correspond to Hi-C domain boundaries.

**Extended Data Fig. 1:**
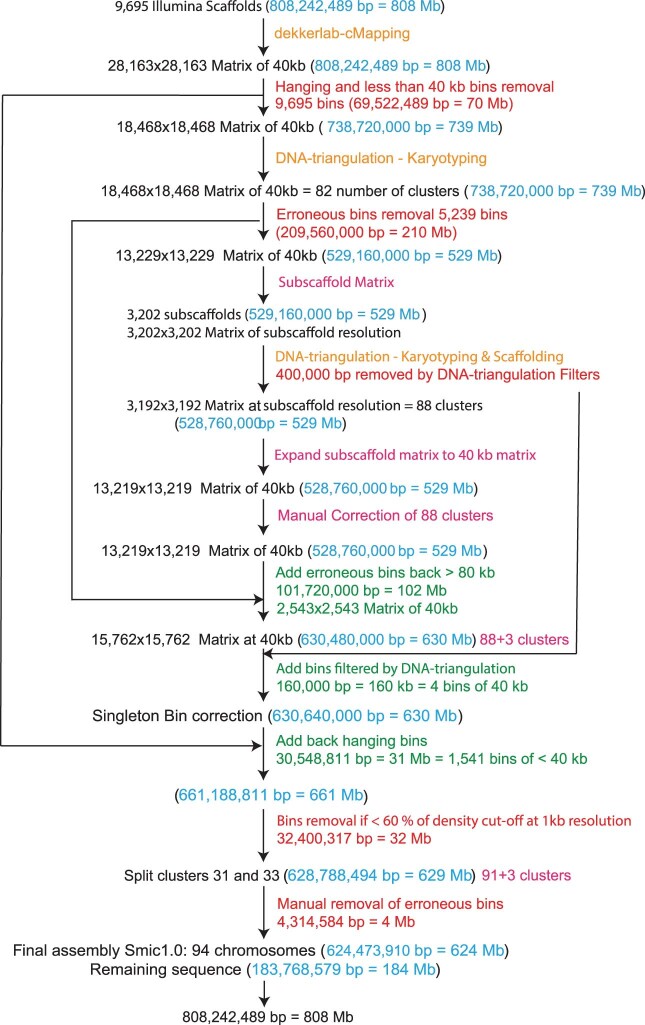
Assembly flow diagram. Diagram of all steps used for Hi-C assisted chromosome-scale assembly of the genome of *S. microadriaticum* (Smic1.0). The flow diagram indicates each step that is described in detail in the Methods section. Red text: steps where sequences were set aside; blue text: the total number of base pairs included in the assembly at the corresponding step; Orange: algorithmic steps using Hi-C read mapping Lajoie et al. Methods 2015 and the DNA triangulation method^27^; green text: number of base pairs placed back into the assembly; purple text: status of the Hi-C interaction matrix and number of chromosomes. A gapfilling step using PacBio reads was used to generate assembly version Smic1.1 N.

Previously, we used Illumina sequencing to build 9,695 scaffolds for *S.* *microadriaticum*^14^. These scaffolds cover 808,242,489 bp (scaffold N50 = 573.5 kb, contig N50 = 34.9 kb). We employed Hi-C^26^ to group, order, and orient these scaffolds into chromosome-scale scaffolds^27,28^. The Hi-C-assisted assembly process is described in the Methods section (Methods, Supplementary Materials and Extended Data Fig. 1) and is summarized in Fig. 1a.

Hi-C reads were mapped to the set of Illumina-based scaffolds (Supplementary Table 1). A total of 2,324,324,062 uniquely mapping chromatin interactions were obtained. Hi-C data were binned at 40-kb resolution and corrected for intrinsic experimental biases^29^. We used hierarchical clustering (karyotyping^27^) to identify groups of bins that interact frequently with each other and are, therefore, likely located on the same chromosome. We identified 94 clusters, representing 94 chromosomes. Subsequently, we used Hi-C-based scaffolding^27^ to order and orient subscaffolds along each chromosome. Subscaffolds are consecutive sections of the original Illumina-based scaffolds that are located on the same chromosome based on Hi-C interaction frequency (Methods).

After extensive manual curation of the assembly using Hi-C data binned at 1-kb resolution, we obtained a genome-wide assembly (Smic1.0) that contains 94 chromosome-scale scaffolds that combined cover 624,473,910 bp (77% of the starting 808,242,489 bp; scaffold N50 = 8.44 Mb, contig N50 = 23.35 kb; Supplementary Table 2). The chromosome number is close to previous estimates of 97 ± 2 chromosomes based on electron microscopic analyses^30–33^. Chromosome lengths range from 26,204 bp to 16,626,072 bp (median = 6,643,339 bp).

### Gap filling and placing higher copy sequences

In the process of assembly, we set aside a total of 183,768,579 bp because they interacted frequently with more than one chromosome. Increased Hi-C interactions between loci located on different chromosomes can occur when sequences are present in several copies at a single location or are present in several locations but are included only once at one location in the assembly. Analysis of Hi-C read coverage indicated that while sequences present on the assembled chromosomes 1–94 are all present at similar copy number, many of the subscaffolds that were set aside were present at much higher copy number (on average 11 times higher than sequences included on the chromosome scaffolds; Fig. 1b). The excluded subscaffolds could be clustered in 94 groups (referred to here as clusters 1–94) based on their Hi-C interaction frequencies. Each of these clusters interacts particularly frequently with only 1 of the 94 chromosomes (Fig. 1b). An overall gene ontology (GO) biological process enrichment analysis showed highly significant overrepresentation of genes associated with DNA integration, reverse transcription, DNA replication and transposition.

**Extended Data Fig. 2:**
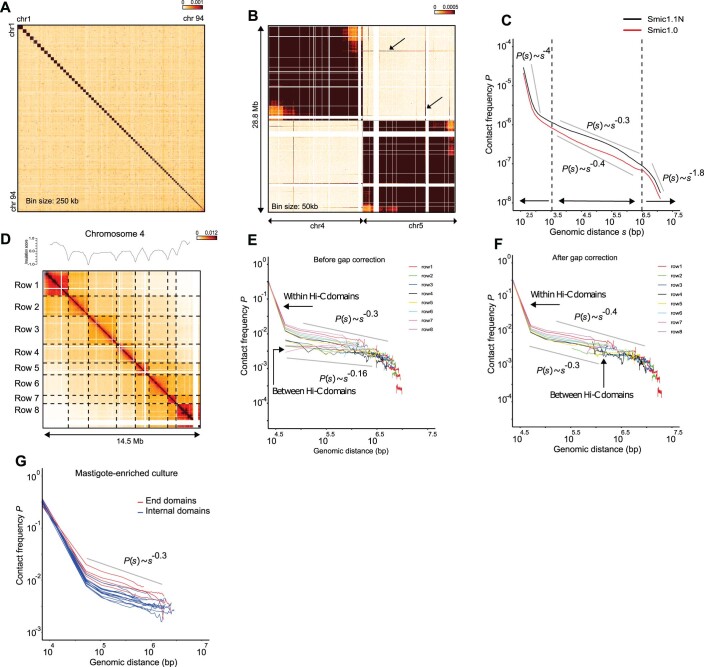
Hi-C data mapped to Smic1.1N. **a**, Hi-C interaction map for the set of 94 chromosomes (ordered by descending size, 250 kb bins) of Smic1.1 N. **b**, Hi-C map for sections of chromosomes 4 and 5, and interactions between chromosome 4 and 5. Arrows indicate line-like features highlighting assembly problems where sequences on chromosome 4 (or 5) interact too frequently with sequences along chromosome 5 (or 4). **c**, Genome-wide contact frequency *P* versus genomic distance *s* for mastigotes-enriched cultures. *P*(*s*) displays three regimes (I, II and III) with distinct exponents (indicated with gray straight lines). Gray curve: Hi-C mapped to Smic1.1 N; red curve: Hi-C data mapped to Smic1.0. **d**, Hi-C interaction map for chromosome 4 (Smic1.1 N; bin size = 50 kb) for mastigotes. Dotted lines indicate domain boundaries and define a set of squares across the interaction map. Plots on top of the heatmaps represent insulation profiles (10 kb resolution, window size 500 kb; see Methods). This profile represents the number of interactions that occur across each location. Local minima in these profiles indicate the locations of sites across which interaction frequencies are relatively low and these correspond to Hi-C domain boundaries. **e**, *P*(*s*) for each square defined by domain boundaries in panel D. Hi-C data from mastigotes. Each individual line represents *P*(*s*) for a single square, colored by row (indicated in panel D). The estimated exponent for *P*(*s*) for regime II ranges from ~-0.16 to -0.3 as indicated by the straight gray lines. Plots for chromatin interaction data within contiguous Hi-C domains, and between Hi-C domains are indicated. **f**, As panel E, but after correcting genomic distances (*s*) for estimated gap sizes between adjacent Hi-C domains. The estimated exponent for *P*(*s*) for regime II is between -0.3 and -0.4 as indicated by the straight gray line. **g**, *P*(*s*) plots for Hi-C domains located at the telomeric ends of chromosomes (red lines) and for domains located internally (blue lines) for chromosomes 4, 26 and 59. Hi-C data obtained with cultures enriched in mastigotes. The *P*(*s*) plots are very similar for coccoid-enriched cultures.

To place these repetitive subscaffolds on the assembled chromosomes, we generated contigs using long reads sequenced on a PacBio RSII instrument (Methods) and aligned these contigs to Smic1.0. Contiguously aligned segments of Smic1.0 were then replaced with the corresponding sections of PacBio contigs. This resulted in extensive gap filling and addition of 111,219,491 bp of sequence to Smic1.0. We found that this process led to the chromosomal placement of ~91 Mb of the ~183 Mb of the sequences that make up the high-copy clusters 1–94 in Smic1.0. We refer to this gap-filled genome as Smic1.1N (Methods and Extended Data Fig. 2a). This assembly covers 735,693,401 bp (scaffold N50 = 9.9 Mb, contig N50 = 467 kb, chromosome sizes range from 27,448 to 19,282,064 bp, median = 7,826,525 bp). However, gap-filling also introduced new assembly errors as evidenced by sequences interacting with several chromosomes (Extended Data Fig. 2b). For analyses described below, we focused on the thoroughly manually curated Smic1.0 assembly, but also performed all analyses on Smic1.1N, which produced nearly identical results (Extended Data).

### Hi-C interaction map has domainal features

**Extended Data Fig. 3:**
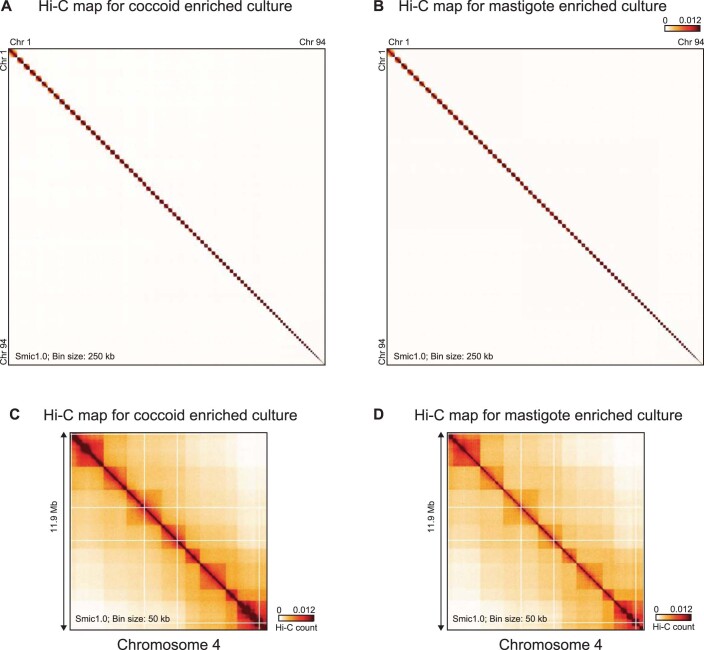
Hi-C interaction maps obtained from coccoid-enriched and mastigote-enriched cultures reveal no obvious differences. **a**, Genome-wide Hi-C interaction map for all 94 assembled chromosomes obtained with a coccoid-enriched culture. Bin size is 250 kb. **b**, Genome-wide Hi-C interaction map for all 94 assembled chromosomes obtained with a mastigote-enriched culture. Bin size is 250 kb. **c**, Hi-C interaction map for chromosome 4 obtained with a coccoid-enriched culture. Bin size is 50 kb. **d**, Hi-C interaction map for chromosome 4 obtained with a coccoid-enriched culture. Bin size is 50 kb.

The Hi-C interaction maps of all chromosomes show domainal features (Fig. 1c): each chromosome has a series of square-shaped domains along the diagonal with relatively elevated interaction frequencies within them and lower frequencies between them. The boundaries between them are often, but not always, sharp transitions. Further, interactions between these Hi-C domains form a series of squares and rectangles farther from the diagonal. Hi-C interaction maps obtained from cultures enriched in coccoid cells (G2/M immobile cells) or mastigote cells (G1/S flagellated cells) showed no obvious differences (Extended Data Fig. 3).

One possible explanation for domain boundaries is the presence of gaps in the genome assembly. However, domain patterns for chromosomes were nearly identical for Smic1.0 and Smic1.1N (for example, compare Fig. 1c with Extended Data Fig. 2d), indicating that most boundaries are resistant to extensive gap filling. To further analyze these domainal features, we used the previously described insulation metric to determine the positions of Hi-C domain boundaries genome-wide at 10-kb resolution (Fig. 1c)^34^. We identified 441 domain boundaries (excluding chromosomes 83–94, which are too short for this analysis). Visual inspection suggests this analysis did not identify some weaker domain boundaries. For Smic1.1N, we identified 468 boundaries, of which 227 were located in contiguous sequence (that is, located at least 10 kb from a contig end). This strongly indicates that boundaries are genuine chromosome structural features and not the result of remaining gaps in the assemblies (see below).

### Two patterns of GC content fluctuations

**Fig. 2:**
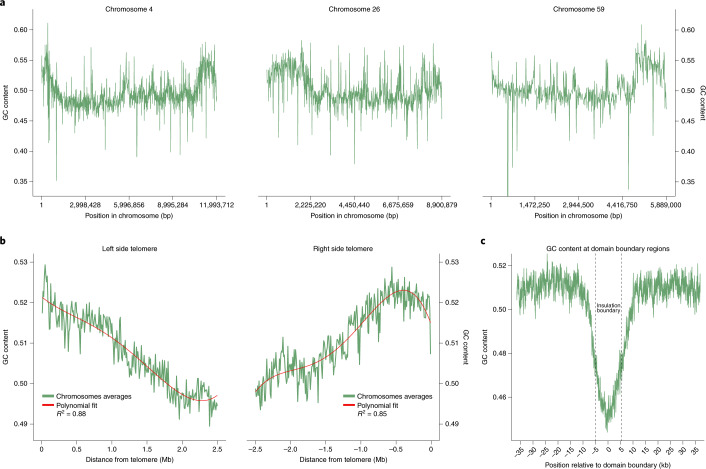
GC content along chromosomes as well as near telomeres and Hi-C domain boundaries for Smic1.0. **a**, GC content fluctuations along chromosomes 4, 26 and 59 measured in 10-kb windows. **b**, GC content along regions 2.5 Mb from telomeric ends, averaged for chromosomes of sizes of at least 5 Mb and measured in 10-kb windows. GC content decreases as distance to telomeres increases. **c**, GC content around domain boundaries. Values are averaged across all domain boundaries in the genome for regions 30 kb upstream and downstream domain boundaries and in 100-bp sliding windows. Dotted lines delimit position of the 10-kb boundaries. A decline in GC content is observed at boundaries that define Hi-C domains.

**Extended Data Fig. 4:**
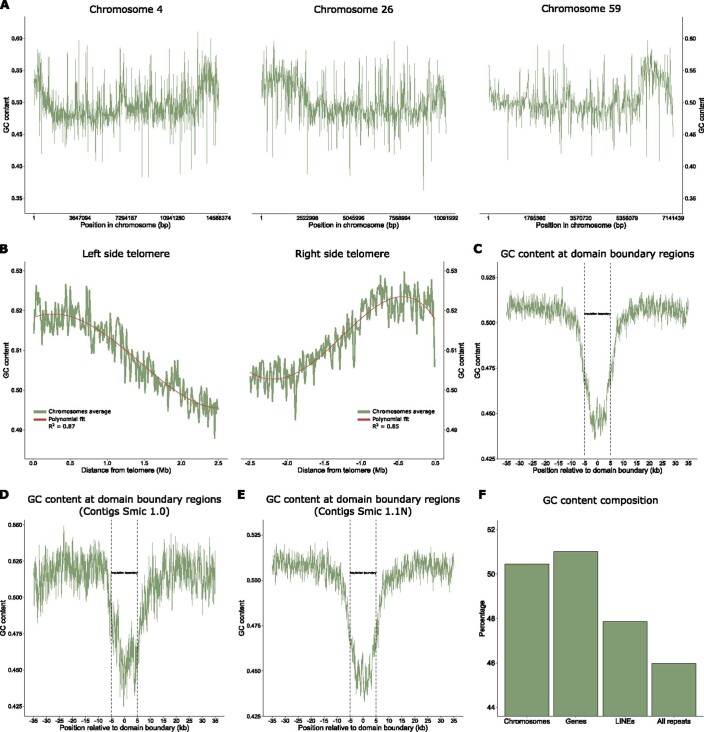
GC content along chromosomes and near telomeres for Smic1.1N, and for Hi-C domain boundaries for Smic1.0 and Smic1.1N. **a**, GC content fluctuations along chromosomes 4, 26, and 59 measured in 10 kb windows for Smic1.1 N assembly. **b**, GC content along regions 2.5 Mb from telomeric ends, averaged for chromosomes of sizes of at least 5 Mb and measured in 10 kb windows for Smic1.1 N. GC content decreases as distance to telomeres increases. **c**, GC content around domain boundaries for Smic1.1 N. Values are averaged across all domain boundaries in the genome for regions 30 kb upstream and downstream domain boundaries and in 100 bp sliding windows. Dotted lines delimit position of the 10 kb boundaries. A decline in GC content is observed at domain boundaries that define Hi-C domains. **d**, GC content around domain boundaries found in ungapped contigs for Smic1.0. This set of boundaries (each a 10 kb region) was located at least 10 kb from a contig end. Values are averaged across all domain boundaries in the genome for 30 kb upstream and downstream domain boundaries and in 100 bp sliding windows. Dotted lines delimit position of the 10 kb boundaries. A decline in GC content is observed at domain boundaries that define Hi-C domains. **e**, GC content around domain boundaries found in ungapped contigs for Smic1.1 N. This set of boundaries (each a 10 kb region) was located at least 10 kb from a contig end. Values are averaged across all domain boundaries in the genome for regions 30 kb upstream and downstream domain boundaries and in 100 bp sliding windows. Dotted lines delimit position of the 10 kb boundaries. A decline in GC content is observed at domain boundaries that define Hi-C domains. **f**, GC content of chromosomes, genes, LINEs, and repetitive elements for Smic1.0. Suggesting that GC content is driven by genes.

We find two chromosome-scale patterns of GC content fluctuations: (1) GC content increases towards the ends of the chromosomes (Fig. 2a,b) and (2) GC content dips to form small local minima at Hi-C domain boundaries (Fig. 2c); the GC content decreases by ~6% from 51 to 46%. The dip in GC content observed at domain boundaries could suggest that this chromosome architectural feature is encoded in the genome. These findings were also true for the gap-filled genome Smic1.1N (Extended Data Fig. 4). Furthermore, considering only Hi-C boundary sequences located in a contig (that is, at least 10 kb away from the boundaries of a contig), we found the same pattern (Extended Data Fig. 4d,e), This provides further evidence that these chromatin domain boundaries detected by Hi-C and their distinct sequence composition are bona fide chromosomal features.

### Some chromosomes are enriched in distinct sets of genes

Genome models from Aranda et al.^14^ were mapped to Smic1.0 (Supplementary Data Files 1–3). Of the 49,109 gene models, 48,715, corresponding to 99% of the genes, were successfully mapped using Minimap2 (ref. ^35^). We used GO term enrichment to investigate whether genes located on the same chromosome were functionally related (Supplementary Data Files 4–6). We also checked for tandem-arrayed genes, with the motivation that genes in such arrays might be linked to related processes. We found genes involved in photosynthesis (chloroplastic), nitrogen cycling and stress response (among others) to be enriched on certain chromosomes. For instance, chromosome 4 contains seven genes of chloroplastic ATP synthase subunit c genes, six of which followed each other in direct vicinity (Smic9977, Smic9979, Smic9980, Smic9981, Smic9983 and Smic9984). Furthermore, chromosome 4 contains 16 genes encoding the pentatricopeptide repeat-containing proteins, organized in three clusters, besides other chloroplastic genes (for example, PsbP domain-containing protein 7, short-chain dehydrogenase TIC 32, etc.). We also found a surplus of tandem-arrayed nitrogen-related genes (12 out of a total of 51) on chromosome 5. Notably, we found clusters of nitrate transporters (*n* = 9 genes arranged in two clusters of 6 and 2 genes, and 1 gene) and nitrate reductases (*n* = 3 genes), some of which were tandem-arrayed, and clusters of Ankyrin repeat domain-containing (*n* = 6 genes) and Ankyrin-2 proteins (*n* = 8 genes). Chromosome 33 has two clusters of ammonia channel/transporter genes, corroborating the view that Symbiodiniaceae feature extensive gene duplication associated with the provisioning of nitrogen^14^. Chromosome 23 is enriched for genes involved in the response to stress, notably signified by a vast expansion of genes annotated as either BTB/POZ and MATH domain-containing protein 2s or BTB and MATH domain-containing protein, similar to chromosome 31, which contains 40 BTB and MATH domain-containing protein genes in two clusters, suggesting expansion of genes encoding these proteins in *S.* *microadriaticum* and showing their presence in a few specific clusters, as found previously for *Arabidopsis* and rice^36^. This chromosome further contains 150 genes of chloroplastic pentatricopeptide repeat-containing proteins in various clusters, putatively involved in RNA editing^37^. Enrichment is driven by the occurrence of the same gene in several copies, and by colocation of different genes involved in the same process on the same chromosome. At present, it is unclear whether the clustering of functionally related genes along certain chromosomes is a simple consequence of the tandem array arrangement of genes due to mechanisms of duplication or whether this is selected for regulatory reasons.

### Gene density increases towards telomeres

**Fig. 3:**
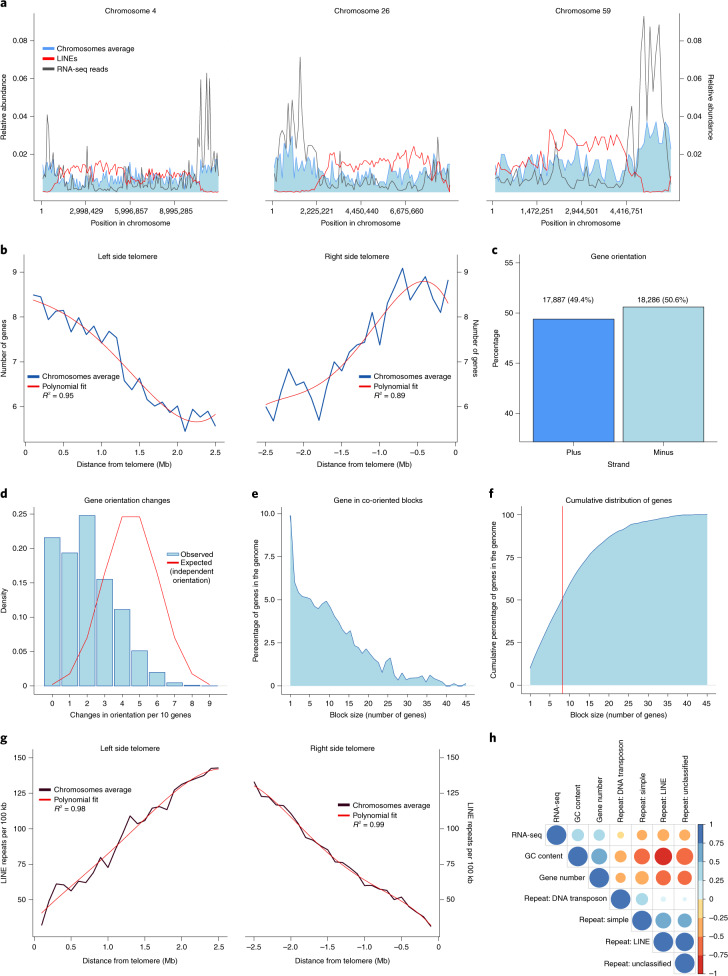
Gene and repetitive element distribution along chromosomes for Smic1.0. **a**, Relative abundance of genes (blue), LINE repeats (red) and mapped RNA-seq reads (gray) for chromosomes 4, 26 and 59. **b**, Gene number along regions 2.5 Mb from telomeric ends, averaged for chromosomes of sizes of at least 5 Mb and measured in 100-kb windows. Gene number is observed to decrease as distance to telomeres increases. **c**, Directionality of genes in the genome. A similar number of genes is found in both strands. **d**, Frequencies of changes in gene orientation. Gene orientation changes defined as the occurrences of neighboring genes located in opposite strands and measured in sliding windows of 10 genes. Observed (blue) and assuming an equal and independent probability of gene orientation (red). **e**, Distribution of genes in blocks of co-oriented genes. **f**, Cumulative distribution of genes in blocks of co-oriented genes; 50% of the genes are found in blocks of 9 or more co-oriented genes (red). **g**, LINEs number along regions 2.5 Mb from telomeric ends, averaged for chromosomes of sizes of at least 5 Mb and measured in 100-kb windows. LINEs number is observed to increase as distance to telomeres increases. **h**, Correlations between gene number, GC content, RNA-seq data and repeat types: LINE, DNA transposons, Simple and Unclassified. Correlation coefficients are shown as a color and size gradient between positive (blue) and negative (red) values. Correlations were done at 100-kb windows.

**Extended Data Fig. 5:**
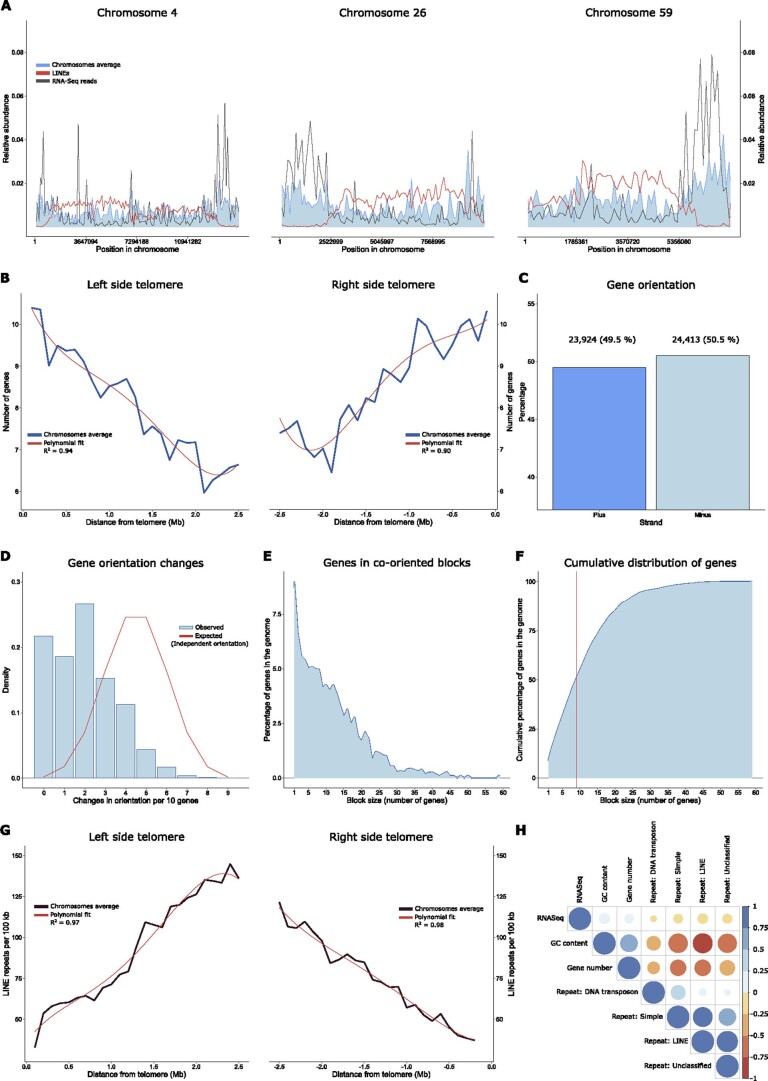
Gene and repetitive element distribution along chromosomes for Smic1.1N. **a**, Relative abundance of genes (blue), LINE repeats (red), and mapped RNA–Seq reads (gray) for chromosomes 4, 26, and 59. **b**, Gene number along regions 2.5 Mb from telomeric ends, averaged for chromosomes of sizes of at least 5 Mb and measured in 100 kb windows. Gene number is observed to decrease as distance to telomeres increases. **c**, Directionality of genes in the genome. A similar number of genes is found in both strands. **d**, Frequencies of changes in gene orientation. Gene orientation changes defined as the occurrences of neighboring genes located in opposite strands and measured in sliding windows of 10 genes. Observed (blue) and assuming an equal and independent probability of gene orientation (red). **e**, Distribution of genes in blocks of co-oriented genes. **f**, Cumulative distribution of genes in blocks of co-oriented genes. 50% of the genes are found in blocks of 9 or more co-oriented genes (red). **g**, LINEs number along regions 2.5 Mb from telomeric ends, averaged for chromosomes of sizes of at least 5 Mb and measured in 100 kb windows. LINEs number is observed to increase as distance to telomeres increases. **h**, Correlations between Gene number, GC content, RNA–Seq data, and Repeat types: LINE, DNA transposons, Simple, and Unclassified. Correlation coefficients are displayed as a color and size gradient between positive (blue) and negative (red) values. Correlations were done at 100 kb windows.

Gene density in *S.* *microadriaticum* ranges from 38 to 155 genes per Mb, showing a greater gene density compared with other eukaryotic genomes such as human (3.5–23 genes per Mb, excluding the Y chromosome) and mouse (7.5–15.9 genes per Mb)^38^, but a gene density comparable with that of *Drosophila* (200 genes per Mb)^39^. Gene density increases towards the telomeres (Fig. 3a,b and Extended Data Fig. 5), having an average gene number of ~9 per 100 kb at the end of the chromosomes and decreasing to ~6 towards the central region (Fig. 3b).

### Genes tend to be organized in unidirectional blocks

The organization and orientation of genes along chromosomes shows an even distribution across strands (Fig. 3c and Extended Data Fig. 5 for Smic1.1N). However, the orientation of neighboring genes is highly correlated and neighboring genes rarely change orientation. Within a 10-gene window, gene orientation changes are strikingly infrequent, similar to our previous analysis^14^ (Fig. 3d and Extended Data Fig. 5). We observe that genes are preferably organized in blocks of co-oriented genes (Fig. 3e), with less than 10% of the genes found without a co-oriented neighbor. Furthermore, 50% of the genes in the genome are organized in blocks of nine or more co-oriented genes (Fig. 3f). Such a pattern, where immediate neighboring genes are more likely to follow the same orientation, is commonly observed in prokaryotes, while in most eukaryotes orientation of neighboring genes is less, or not, correlated. Notable exceptions include kinetoplastids, which also show blocks of unidirectional genes^40^.

### Chromosomal distribution of repetitive elements

The most abundant repetitive elements in *S.* *microadriaticum* are long interspersed nuclear elements (LINEs), followed by simple repeats, unclassified repeats and DNA transposons, constituting 13.36, 5.79, 4.61 and 1.56% of the genome, respectively.

Repetitive elements follow the opposite pattern to gene density, expression and GC content, being lower towards the ends of the chromosomes (Fig. 3a,g,h and Extended Data Fig. 5). The locations of LINE elements are correlated positively with the presence of other repetitive elements, indicating that repetitive elements are, in general, enriched in the middle of the chromosomes (Fig. 3g).

In summary, our analysis shows that gene density and gene expression are generally higher towards chromosome ends, resulting in a moderate, yet significant, positive correlation between gene density and gene expression (*R*^2^ = 0.33, *P* = 4.2 × 10^−157^) (Fig. 3a,h and see Extended Data Fig. 5 for this analysis for Smic1.1N). Furthermore, gene density and expression were correlated positively with GC content (Fig. 3h and Extended Data Fig. 5), and negatively with repeat density. This is in line with the notion that high GC content regions are associated with gene-rich regions.

### Chromosomes are folded as linearly organized rods

**Fig. 4:**
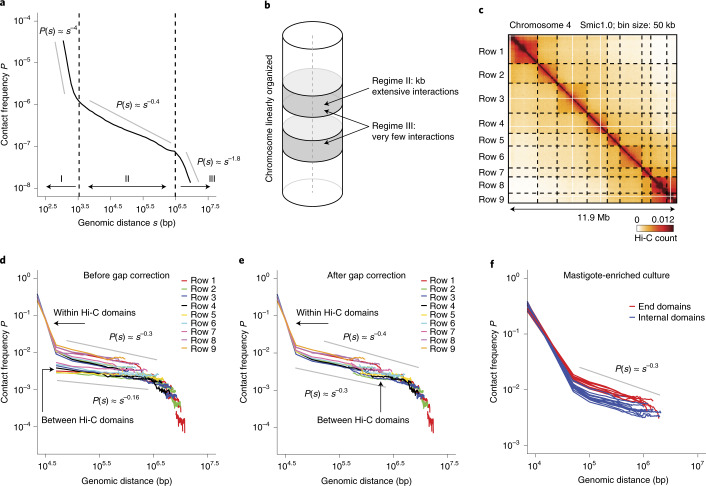
Linear organization of *S.* *microadriaticum* chromosomes. **a**, Genome-wide contact frequency *P* versus genomic distance *s* for mastigote-enriched cultures. *P*(*s*) shows three regimes (I, II and III) with distinct exponents (indicated with gray straight lines). The *P*(*s*) plot is very similar for coccoid-enriched cultures. **b**, Schematic depiction of a linearly organized chromosome. Linear organization is predicted from the presence of the steep drop in *P*(*s*) (regime III). The shallow decay in interaction frequency followed by a steep drop at ~3 Mb is consistent with an organization where loci have interactions only with loci located within a limited genomic distance (~3 Mb up- and downstream). Regime I is, at least in part, due to the presence of aberrant Hi-C molecules known to occur at very short distances (unligated ends and self-circularized fragments; Extended Data Fig. 6). **c**, Hi-C interaction map for chromosome 4 (bin size = 50 kb) for mastigotes. Dotted lines indicate domain boundaries and define a set of squares across the interaction map. **d**, *P*(*s*) for each square defined by domain boundaries in (**c**). Hi-C data from mastigotes. Each individual line represents *P*(*s*) for a single square, colored by row (indicated in panel C). The estimated exponent for *P*(*s*) for regime II ranges from around −0.16 to −0.3 as indicated by the straight gray lines. Plots for chromatin interaction data within contiguous Hi-C domains, and between Hi-C domains are indicated. **e**, As **d**, but after correcting genomic distances (*s*) for estimated gap sizes between adjacent Hi-C domains. The estimated exponent for *P*(*s*) for regime II is between −0.3 and −0.4, as indicated by the straight gray line. **f**, *P*(*s*) plots for Hi-C domains located at the telomeric ends of chromosomes (red lines) and for domains located internally (blue lines) for chromosomes 4, 26 and 59. Hi-C data obtained with cultures enriched in mastigotes. The *P*(*s*) plots are very similar for coccoid-enriched cultures.

**Extended Data Fig. 6:**
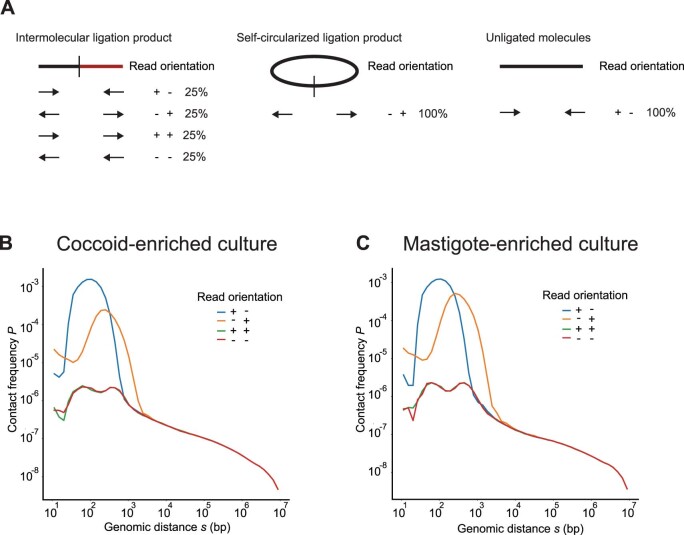
*P*(*s*) plots for different Hi-C read orientations. **a**, Different types of Hi-C ligation products. Ligation of two interacting restriction fragments can occur in 4 different fragment orientation (head-to-head, tail-to-tail, head-to-tail and tail-to-head). These 4 products can be identified by the Hi-C read orientation, as indicated, and are expected to occur with equal frequency (25% for each type). Self-circularized (partial) digestion products, or unligated linear fragments are also produced in Hi-C and are uninformative. These two products are characterized by the two Hi-C read orientations for each molecule: (-) and (+) for self-circularized molecules and (+) and (-) for unligated molecules. **b**, Genome-wide *P*(s) plots for each of the 4 Hi-C read orientations for coccoid-enriched cultures. For distances below 2–3 kb uninformative Hi-C dominate, while for genomic distance over 2–3 kb all 4 read-orientation occur with equal frequency. Hi-C data was mapped against Smic1.0. **c**, Genome-wide *P*(s) plots for each of the 4 Hi-C read orientations for mastigote-enriched cultures. For distances below 2–3 kb uninformative Hi-C dominate, while for genomic distance over 2–3 kb all 4 read-orientations occur with equal frequency. Hi-C data was mapped against Smic1.0.

Next, we leveraged the Hi-C data to obtain insights into the spatial organization of *S.* *microadriaticum* chromosomes. When average interaction frequency *P* is plotted as a function of genomic distance *s* (*P*(*s*)), a general inverse relationship is typically observed and, from the shape and exponent of the curve, features of chromosome folding can be inferred. We plotted *P*(*s*) for *S.* *microadriaticum* (Fig. 4a). The shape of *P*(*s*) suggests three regimes. First, for loci separated by a few kilobases there is a very steep decay. Read orientation analysis shows that the steep decay in regime I is the result of noninformative Hi-C ligation products^41^ (Extended Data Fig. 6). Second, for loci separated by several kilobases up to ~3 Mb there is a very shallow decay (*P*(*s*) ≈ *s*^−0.4^). Third, for loci separated by more than ~3 Mb there is a steep drop in contact frequency. The overall shape of *P*(*s*) is reminiscent of that observed for mitotic chromosomes in vertebrates. We have previously demonstrated that such *a P*(*s*) shape is consistent with the formation of rod-shaped chromosomes^42,43^. This is consistent with microscopic observations of chromosomes in a variety of dinoflagellates that show elongated rod-shaped and permanently condensed chromosomes (for example, ref. ^44^). The steep drop in interaction frequency for loci separated by more than ~3 Mb indicates that, along the long-axis of the stiff rod-shaped chromosome, loci rarely interact with loci located more than 3 Mb away along the linear genome (Fig. 4b). All chromosomes, regardless of their length, show the steep drop in *P*(*s*) at ~3 Mb, which indicates that the internal organization of chromosomes is independent of chromosome length.

The exponent of *P*(*s*) for regime II shows some properties of how chromatin is organized in a cross-section of the rod-shaped chromosome^42,43^. For *S.* *microadriaticum*, the exponent of *P*(*s*) in the intralayer regime is small: based on the global *P*(*s*) plot shown in Fig. 4a, the exponent is close to ~0.4. For Smic1.1N the exponent is somewhat smaller, between −0.3 and −0.4 (Extended Data Fig. 2). We also plotted *P*(*s*) for interactions that occur in individual Hi-C domains, excluding any interactions that occur across Hi-C domain boundaries (Fig. 4c,d and Extended Data Fig. 2). The exponent of *P*(*s*) for interactions in individual domains is consistently around −0.3 (Fig. 4d,e). Such small exponent indicates extensive packing and potential mixing of DNA in cross-sections of the rod-shaped chromosomes.

We observe more variable exponents when we plotted *P*(*s*) for interactions between Hi-C domains; exponents ranged from −0.1 to −0.4. When we assume that domain formation is due entirely to sequence gaps, though highly unlikely (see above and below), the *P*(*s*) plots in Fig. 4d can be used to estimate the size of such putative gaps by determining how much individual interdomain *P*(*s*) plots need to be shifted along the x-axis so that they all overlap (Methods). In most cases, plots would need to be shifted several hundred kilobases. After such putative gap correction, the estimated exponents for regime II for the different sections of the Hi-C map again ranged between −0.3 and −0.4 (Fig. 4e). Nearly identical results are found when *P*(*s*) was analyzed for Hi-C data mapped to Smic1.1N (Extended Data Fig. 2).

### Fluctuation of chromatin compaction along chromosomes

Visual inspection of Hi-C interaction maps shows that interactions tend to be of higher frequency near the ends of all chromosomes. To quantify this, we plotted *P*(s) for telomeric Hi-C domains and for internally located Hi-C domains (Fig. 4f). We observed that chromatin interactions in terminal domains are about twofold higher for loci separated up to 1 Mb, while the exponent of *P*(*s*) is very similar for all domains (around −0.3). One interpretation is that the chromatin fiber has a shorter contour length near the telomeric ends as compared with chromatin in the middle portions of the chromosomes^45^. Very similar results were obtained when Hi-C data was mapped to Smic1.1N (Extended Data Fig. 2).

### Domain boundaries occur at sites where genes converge

**Fig. 5:**
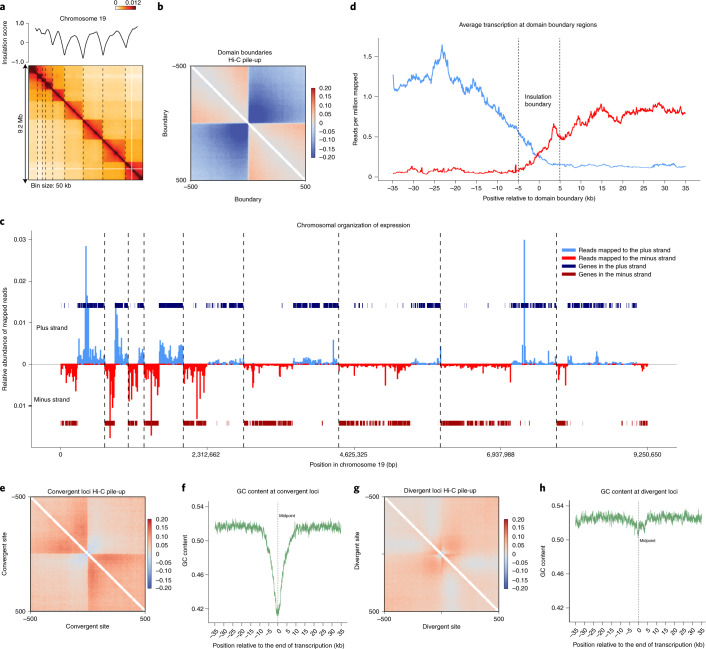
Correlation between Hi-C domains and unidirectional gene blocks. **a**, Hi-C interaction map for chromosome 19 (Smic1.0; bin size = 50 kb) for mastigotes. Dotted lines indicate domain boundaries. Plot on top of the heatmap represents the insulation profile (10-kb resolution, window size 500 kb). **b**, Average Hi-C interactions around the set of 441 domain boundaries (±500 kb) at 10-kb resolution. Data are log_10_(observed interaction frequency/expected interaction frequency). **c**, Chromosome 19 transcription and domain landscape. Indicated are: transcripts mapping to the plus strand (light blue), transcripts mapping to the minus strand (red), genes on the plus strand (dark blue), genes on the minus strand (dark red), and domain boundaries (dotted vertical lines). **d**, Average relative transcription around boundaries. Values are averaged across all domain boundaries in the genome for regions 30 kb upstream and downstream domain boundaries and in 100-bp sliding windows. Dotted lines delimit domain boundary, which is a 10-kb region based on Hi-C data. The limited amount of transcription observed to proceed through the boundary can be the result of the imprecision of boundary detection (10 kb). **e**, Average Hi-C interactions around 280 manually curated convergent sites (±500 kb), previously not identified as domain boundaries, at 10-kb resolution. Data are log_10_(observed interaction frequency/expected interaction frequency). **f**, GC content around all convergent sites. Values are averaged across all manually curated convergent sites in the genome for regions 35 kb upstream and downstream to the midpoint between two expression blocks (dotted line) in 100-bp sliding windows. Data are log_10_(observed interaction frequency/expected interaction frequency). **g**, Average Hi-C interactions around 517 manually curated divergent sites (±500 kb) at 10-kb resolution. Data are log_10_(observed interaction frequency/expected interaction frequency). **h**, GC content around divergent sites. Values are averaged across all manually curated divergent sites in the genome for regions 35 kb upstream and downstream to the midpoint between two expression blocks (dotted line) in 100-bp sliding windows.

We next investigated the relationship between gene orientation, transcription and features of chromosome conformation observed with Hi-C. Figure 5a shows the Hi-C interaction map for chromosome 19, with domain boundaries indicated by dotted lines.

The sharp boundaries in chromatin interactions are readily detected when we aggregate Hi-C interactions around boundaries genome-wide (Fig. 5b); interactions across domain boundaries are depleted. To determine whether there is a relationship between unidirectional gene blocks and chromosomal domains, we plotted RNA expression along each chromosome in a strand-specific manner to highlight blocks of co-expressed co-oriented genes (Fig. 5c). As expected, blocks of transcripts are observed that alternate between being encoded on the top and the bottom strand. Intriguingly, most domain boundaries observed by Hi-C are located at positions where transcription of blocks of unidirectional genes converges. A similar pattern was observed along all chromosomes. To quantify this pattern genome wide, we plotted the number of reads derived from each strand as a function of distance up or downstream of Hi-C domain boundaries (Fig. 5d). We find that reads upstream of a boundary map almost exclusively to the top strand, while reads downstream of a boundary map mostly to the bottom strand.

We did not identify domain boundaries at all locations where transcription of blocks of unidirectional genes converge. This is most likely due to the fact that the parameters we chose for the insulation analysis to identify domains boundaries^34^ are conservative (Fig. 1 and Methods). Visual inspection of Hi-C interaction maps confirms the presence of domain boundaries at most of the locations where gene expression blocks converge. To explore this in another way, we identified all sites where transcription of blocks of co-oriented genes converges. This set includes 388 out of the 441 domain boundaries and a further set of 280 convergent sites that did not overlap a called domain boundary. When we aggregated average Hi-C interactions around this set of further convergent sites, we again observe the formation of a distinct structural boundary (Fig. 5e). We conclude that domain boundaries occur at the large majority of sites of convergent unidirectional gene blocks. In a small minority of cases we detected a domain boundary away from such convergent sites (53 out of 441 boundaries). These could be due to remaining gaps in the assembly, or represent different types of structural boundaries.

**Extended Data Fig. 7:**
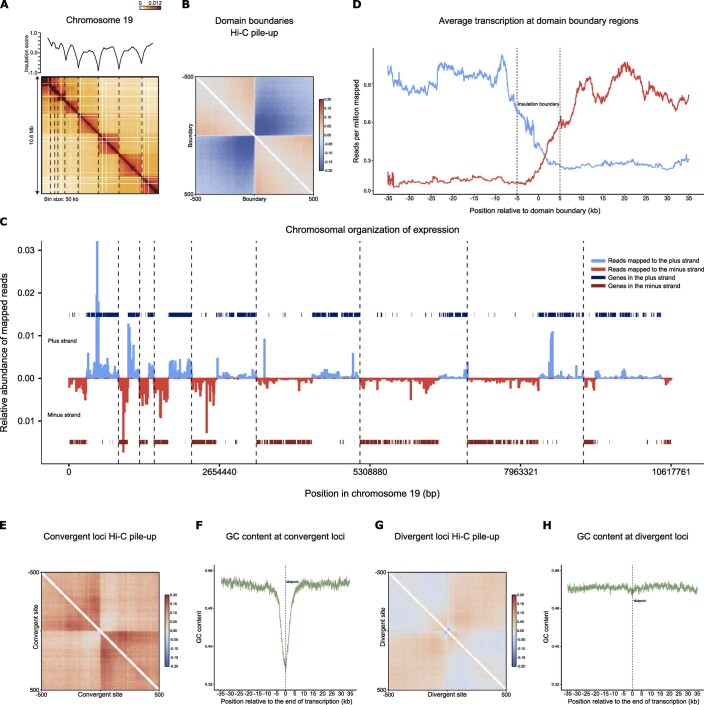
Correlation between Hi-C domains and unidirectional gene blocks for Smic1.1N. **a**, Hi-C interaction map for chromosome 19 (Smic1.1 N; bin size = 50 kb) for mastigotes. Dotted lines indicate domain boundaries. Plot on top of the heatmap represents the insulation profile (10 kb resolution, window size 500 kb; see Methods). **b**, Average Hi-C interactions around the set of 468 domain boundaries at 10 kb resolution. **c**, Chromosome 19 transcription and domain landscape. Indicated are: transcripts mapping to the plus strand (light blue), transcripts mapping to the minus strand (red), genes on the plus strand (dark blue), genes on the minus strand (dark red), and domain boundaries as dotted vertical lines. A clear domainal gene block organization is observed and is delimited by Hi-C domain boundaries. Each domain is a pair of divergent gene blocks. Domain boundaries occur where gene blocks converge. **d**, Average relative transcription around boundaries. Values are averaged across all domain boundaries in the genome for regions 30 kb upstream and downstream domain boundaries and in 100 bp sliding windows. Dotted lines delimit domain boundary, which is a 10 kb region based on Hi-C data. The limited level of transcription that is observed to proceed through the boundary can be the result of the imprecision of boundary detection (10 kb). **e**, Hi-C pile-up plot for 213 manually curated convergent sites, previously not identified as domain boundaries, at 10 kb resolution. **f**, GC content around all convergent sites. Values are averaged across all manually curated convergent sites in the genome for regions 35 kb upstream and downstream to the midpoint between two expression blocks (dotted line) in 100 bp sliding windows. A sharp decline in GC content is observed at convergent regions. **g**, Hi-C pile-up plot for 358 manually curated divergent sites at 10 kb resolution. **h**, GC content around divergent sites. Values are averaged across all manually curated divergent sites in the genome for regions 35 kb upstream and downstream to the midpoint between two expression blocks (dotted line) in 100 bp sliding windows. Contrary to convergent sites, only a slight decline, if any, in GC content is observed at divergent sites.

We aggregated Hi-C interactions around the sites from which divergent unidirectional gene blocks are transcribed within each domain (referred to as the bidirectional locus) (Fig. 5g). We observe that, on average, at bidirectional loci the Hi-C interaction map shows a local boundary; interactions between loci located up to ~100 kb upstream and ~100 kb downstream of the bidirectional locus are depleted. Compared to convergent sites, this boundary effect is much weaker and occurs over only relatively short genomic distances. In addition, we observe lines of enriched interactions that form a ‘plus’ sign. This can represent long-range looping interactions anchored at the bidirectional locus and other loci located at varying distances either up- or downstream. We conclude that both the convergent and divergent sites have specific higher order chromosome structures detected by Hi-C, with the convergent sites forming very prominent boundaries, and the divergent loci minor and locally acting boundaries. A clear reduction in GC content (~10%) is observed only at convergent boundaries (Fig. 5f,h). All results shown in Fig. 5 were reproduced when we repeated the analysis using Smic1.1N (Extended Data Fig. 7).

### Loss of domains in cells treated with transcription blockers

**Fig. 6:**
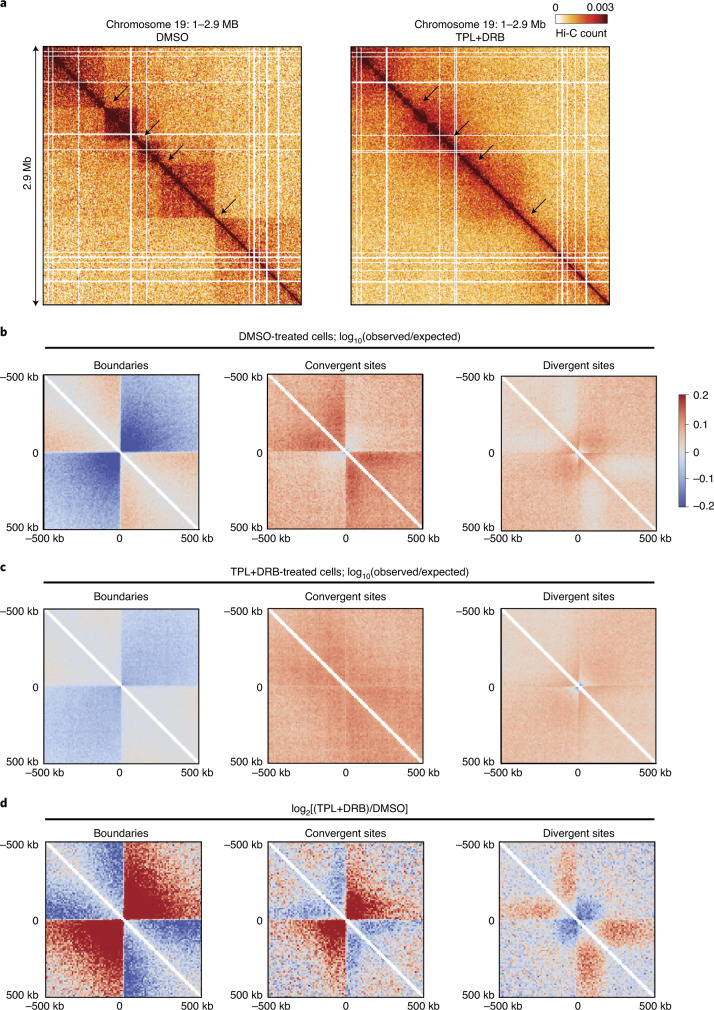
Altered chromosome conformation in cells treated with triptolide and DRB. **a**, Hi-C interaction maps for a section of chromosome 19 (Smic1.0; bins size 10 kb) for cells treated with DMSO (control, left) or triptolide (TRP) and DRB (right). Cells were treated with 25 μM TRP + 50 μM DRB or 200 μM TRP + 400 μM DRB. Both treatments produced similar results, and Hi-C data obtained with the two treatments were pooled. Arrows indicate positions of domain boundaries that are visible in control cells and that disappear in cells treated with triptolide and DRB. **b**, Hi-C data (observed/expected) for DMSO-treated cells, aggregated around domain boundaries (±500 kb; *n* = 441, left panel), convergent sites (*n* = 280; middle panel) and divergent sites (*n* = 517; right panel). Bin size: 10 kb. Data are log_10_(observed interaction frequency/expected interaction frequency). **c**, Hi-C data (observed/expected) for triptolide + DRB-treated cells, aggregated around domain boundaries (±500 kb; *n* = 441, left panel), convergent sites (*n* = 280; middle panel) and divergent sites (*n* = 517; right panel). Bin size: 10 kb. Data are log_10_(observed interaction frequency/expected interaction frequency). **d**, Change in chromatin interactions around domain boundaries (±500 kb; *n* = 441, left panel), convergent sites (*n* = 288; middle panel) and divergent sites (*n* = 517; right panel). Shown is log_2_[(TRP + DRB)/DMSO]. Bin size: 10 kb.

**Extended Data Fig. 8:**
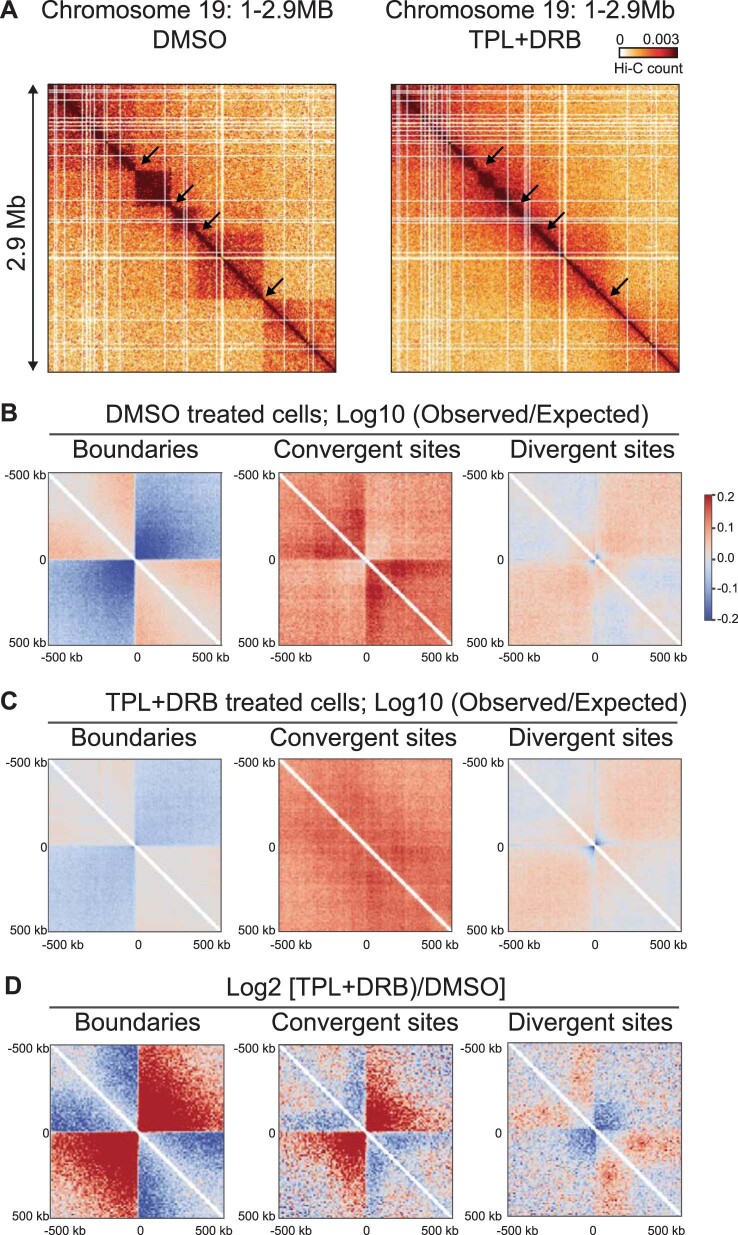
Altered chromosome conformation in cells treated with triptolide and DRB, Hi-C data mapped to Smic1.1N. **a**, Hi-C interaction maps for a section of chromosome 19 (Smic1.1 N; bins size 10 kb) for cells treated with DMSO (control, left) or triptolide (TRP) and DRB (right). Cells were treated with 25 μM TRP + 50 μM DRB, or 200 μM TRP + 400 μM DRB. Both treatments produced similar results, and Hi-C data obtained with the two treatments were pooled. Arrows indicate positions of domain boundaries that are visible in control cells, and that disappear in cells treated with triptolide and DRB. **b**, Hi-C data (observed / expected) for DMSO-treated cells, aggregated around domain boundaries (+/- 500 kb; n = 468, left panel), convergent sites (n = 213; middle panel) and divergent sites (n = 358; right panel). Bin size: 10 kb, Data is log10 (Observed interaction frequency/expected interaction frequency). **c**, Hi-C data (observed / expected) for triptolide+DRB-treated cells, aggregated around domain boundaries (+/- 500 kb; n = 468, left panel), convergent sites (n = 213; middle panel) and divergent sites (n = 358; right panel). Bin size: 10 kb, Data is log10 (Observed interaction frequency/expected interaction frequency). **d**, Change in chromatin interactions around domain boundaries (+/- 500 kb; n = 468, left panel), convergent sites (n = 213; middle panel) and divergent sites (n = 358; right panel). Shown is log2 [(TRP + DRB)/DMSO]. Bin size: 10 kb.

The strong correlation between gene orientation and boundary position suggests a mechanistic relationship between transcription and chromatin domain formation. To test this, we treated cells with chemicals that are known to block transcription in other eukaryotes. We incubated cells for 12 h with combinations of two different concentrations of triptolide (25 µM or 200 µM), and dichlorobenzimidazole 1-β-d-ribofuranoside (DRB; 50 µM or 400 µM), or with dimethylsulfoxide (DMSO; control treatment). This treatment led to growth arrest but not cell death as cultures resumed growth with a normal doubling rate after the chemicals were washed away. We then performed Hi-C on treated and control cultures. We find that while domain boundaries are readily detected in DMSO-treated cells, when cells are treated with either dose of triptolide + DRB, domain boundaries became visibly weaker or disappeared entirely (Fig. 6a). Similar results were found when Hi-C data was mapped to Smic1.1N (Extended Data Fig. 8).

To quantify this effect, we aggregated Hi-C data around Hi-C domain boundaries at sites of convergent transcription and at sites of divergent transcription (as described above). We detect depletion of chromatin interactions across domain boundaries and convergent sites in DMSO-treated cells (Fig. 6b), and this depletion was diminished for cells treated with triptolide and DRB (Fig. 6c,d). We also detected changes in chromatin interaction patterns around sites of divergent transcription: in DMSO-treated cells, characteristic local depletion of interactions and line-like features are observed (as in Fig. 5g). In cells treated with triptolide and DRB, these features change, with a reduction in short-range interactions at the divergent sites, and an increase in longer-range interactions (Fig. 6c,d).

These observations indicate that chromatin conformation is sensitive to treatment of cells with triptolide and DRB. We were not able to identify conditions where *S.* *microadriaticum* takes up modified bases, such as 5-ethynyl uridine (EU), and therefore we were not able to ascertain that this treatment indeed blocks nascent transcription. Based on our data, we can conclude that chromosome conformation is modulated under conditions that block cell growth, possibly through effects on transcription. The fact that domain formation is sensitive to growth conditions shows that domain boundaries are not due to remaining genome assembly errors.

## Discussion

We present a chromosome-scale assembly of the genome of the dinoflagellate *S.* *microadriaticum*. This assembly shows the organization of the genetic information and, together with Hi-C data, shows insights into the spatial organization of chromosomes in this representative of the unique dinoflagellates. We find that genes are enriched near the telomeric ends and are generally arranged in alternating unidirectional blocks. Chromosomes fold in a series of domains, with each domain containing a pair of divergently transcribed gene blocks and domain boundaries located where unidirectional gene blocks converge. This observation suggests a close relationship between gene orientation, gene expression and chromatin domain formation, which was further supported by our observation that domain formation was found to be sensitive to treatment with triptolide and DRB. Similar results were described recently for a different dinoflagellate species^46^. Hi-C data confirm that chromosomes form relatively stiff rod-shaped structures, consistent with previous extensive microscopic observations. In contrast to many other eukaryotes, we did not detect evidence for chromosome compartmentalization in active and inactive spatial compartments. It is possible that the formation of stiff rods prevents long-range compartmental interactions. We did not detect any locus-specific point-to-point looping interactions despite collecting more than 2 billion chromatin interactions for this ~0.8 Gb genome. Line-like features in Hi-C maps at divergent sites suggest that loop extrusion events, anchored at these sites, may occur, consistent with the fact that *S.* *microadriaticum* expresses cohesin and condensin complexes. Such loops may be related to microscopic observations of loops in dinoflagellates^44,47–49^.

**Extended Data Fig. 9:**
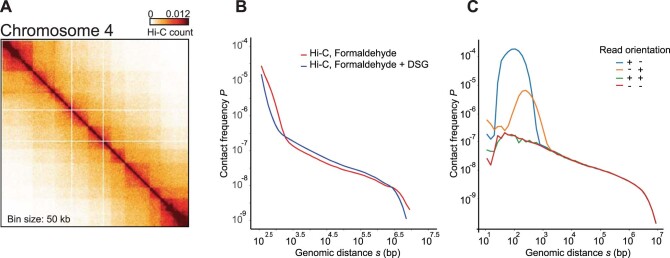
More extensive crosslinking does not greatly alter Hi-C maps and chromatin interaction frequencies. **a**, Hi-C interaction map for chromosome 4 (Smic1.0; bin size = 50 kb; Smic1.0) for coccoid-enriched cultures fixed with formaldehyde and DSG. **b**, Genome-wide contact frequency *P* versus genomic distance *s* for coccoid-enriched cultures fixed with formaldehyde only, or with formaldehyde and DSG. *P*(*s*) displays three regimes (as in Fig. 4a), and the middle section of the plots have similar small exponents. Hi-C data was mapped against Smic1.0. **c**, Genome-wide contact frequency *P* versus genomic distance *s* for coccoid-enriched cultures fixed with formaldehyde and DSG for each of the 4 possible orientation of ligation products. Ideally all 4 orientations display identical *P*(*s*). The + - and - + orientations deviate strongly for s < 2 kb and represent Hi-C artifacts (unligated ends and self-circularized molecules respectively. Hi-C data was mapped against Smic1.0.

Various models have been proposed on how DNA is organized in dinoflagellate chromosomes. One principal hallmark of dinoflagellate chromatin is the observation that most of their DNA is not wrapped around nucleosomes. Histones are replaced by other basic proteins, for example, histone-like proteins derived from bacteria and DVNPs derived from viruses^19–21^. Microscopically, these chromosomes appear as permanently condensed rods, with some variation during the cell cycle^44^. Our Hi-C data are fully consistent with these observations. In one model, this rod-shaped structure represents a helically coiled toroidal chromonema^50^. Our data do not support this model; first, the model assumes helical folding, but our Hi-C maps do not show such features, which would lead to periodic features in interaction maps, for example, as seen in prometaphase chromosomes in chicken cells^43^. Further, this model assumes circular chromosomes, which is not observed by Hi-C. The optical birefringent properties of dinoflagellate chromatin^51,52^ suggest that the DNA has liquid-crystalline features. This has led to a model where the chromosomes fold as cholesteric liquid crystals^22,53–56^. Polymer simulations may provide insights into whether our Hi-C data, and especially the exponent of *P*(*s*) plot, are consistent with liquid-crystalline folding or not. For loci separated by up to 3 Mb, we find that their interaction frequency decays very slowly with increasing genomic distance. The exponent of this decay is around −0.4, and even smaller when *P*(*s*) is analyzed in domains (−0.3), which is much smaller than what is observed in other eukaryotes and even smaller than for mammalian mitotic chromosomes. We considered the possibility that the small exponent we observed was the result of inefficient formaldehyde crosslinking of chromatin interactions in dinoflagellates. Therefore, we also performed Hi-C using a combination of formaldehyde and disuccinimidyl glutarate (DSG). The Hi-C interaction maps and *P*(*s*) plots obtained this way are very similar (Extended Data Fig. 9), suggesting the small exponent is not due to low crosslinking efficiency. The small exponent suggests a very high amount of compaction but does not by itself show how DNA is packed in such layers. Finally, the relationships between the Hi-C domains observed here and microscopically observed structures, such as the banding pattern along liquid-crystalline chromosomes and decondensed loops emanating from the condensed core^48,57,58^, remain to be explored.

Mammalian genomes also have domainal features (topologically associating domains or TADs^59,60^) that superficially resemble the domains we observe here. However, in mammals, TADs do not show a correlation with gene orientation. In yeast, small chromosomal domains have been observed that often have boundaries at convergent genes. In *Drosophila*, a correlation between domain boundaries, gene density and orientation was observed^61^. Trypanosomes also form unidirectional gene blocks but, in contrast to what we observe for *S.* *microadriaticum*, no correlation between gene orientation and domain formation was described^62^. The domains we observe here resemble those seen in the prokaryote *Caulobacter*^63^; they have sharp boundaries, show a similar nested pattern and no boundary loops. In *Caulobacter*, domain boundaries are positioned at highly expressed genes and depend on transcription. Assuming a supercoiled bacterial chromosome, polymer simulations had indicated that domain boundaries can form at sites that block diffusion of supercoils^63^. However, no relation with gene orientation was reported. Future studies are required to test such models or to show alternative mechanisms of domain formation in dinoflagellates.

Many of the peculiarities of Symbiodiniaceae genomes, such as the unidirectional blocks of genes, the high number of genomic genes, the high density of genes or the tandem array arrangement of genes (among others) could be corroborated here. In particular, the observation that a portion of the chromosomes are enriched for specific biological processes (for example, photosynthesis, nitrogen cycling and stress response) and that functionally related genes tend to co-occur at adjacent sites in the genome, add a previously unobserved amount of chromosomal functionalization. Notably, in most cases, we found duplications of specific genes (rather than sets of genes encoding for entire pathways), which, in the course of the GO enrichment analysis, are portrayed as pathway enrichments. While this is a limitation of our analysis, it reflects the notion that specific genes of specific pathways are enriched. From an adaptation perspective, such a structural organization provides the opportunity for dynamic environmental adaptation through chromosome duplication or loss. Varying chromosome counts and polyploidy have been described for field and cultured specimens^64^.

The description of the genetic and spatial organization of the chromosomes of *S.* *microadriaticum*, and that of a different dinoflagellate *Breviolum minutum*^46^, will open new lines of research into the mechanisms of chromosome folding in this extraordinary group of organisms. In addition, given the ecological importance of dinoflagellates, the chromosome-scale assembly of their genomes will be instrumental into explaining their unique biology.

## Methods

### *Symbiodinium microadriaticum* culturing

*S.* *microadriaticum* (clade A) cultures were obtained from the Gulf of Aqaba near Asia, vendor NCMA (National Center for Marine Algae and Microbiota), Bigelow Laboratory for Ocean Sciences (CCMP2467-SC) and grown in F/2 FSW (fresh seawater from the gulf of Maine, VENDOR). Four single colonies of *S.* *microadriaticum* isolated from the original reef sample by growth on F/2 FSW agar plates (clones referred to as D1, D3, D4 and D7) were picked and then continued in liquid medium in the presence of a 1:200,000 dilution from a 50× antibiotics stock (100 ml of 50× antibiotic stock solution contains 5.0 g of penicillin-G,10.0 g of streptomycin, 5.0 g of kanamycin, 1.0 g of neomycin, 75 mg of nystatin, 30 mg of erythromycin, 40 mg of gentamicin, 80 mg of polymyxin-B, 60 mg of tetracycline, 60 mg of vancomycin). Cultures are grown in T75 tissue culture flasks at 23 °C with a 12 h/12 h light/dark cycle, with a light intensity of 60–80 μE m^−2^ s^−1^. Once a week, cultures were split by first removing the supernatant and adding fresh medium 2.5 h before the start of the light phase. The medium with newly born mastigotes is transferred to a new flask 3 h later (0.5 h after the light phase has started) and the old vessel is discarded.

### Triptolide and DRB treatment conditions

For triptolide (Millipore, catalog no. 645900-5MG) and dichlorobenzimidazole DRB (Sigma, catalog no. D1916-50MG) experiments, newborn cD3+ mastigotes were treated either with triptolide with DRB at 25 µM and 200 µM, or triptolide with DRB at 50 µM and 400 µM as the final concentrations. Control mastigotes were treated with DMSO. Cells were harvested after 22 h treatment and crosslinked with 1% formaldehyde with 3 mM DSG as the final concentration. Hi-C was carried out as described below.

### Hi-C procedure

We adapted the conventional Hi-C protocol for analysis of *S.* *microadriaticum* chromosomes and obtained Hi-C datasets for cultures enriched in mastigotes, and for cultures enriched in coccoid cells (Supplementary Methods). For initial assembly, we pooled all Hi-C data (four replicates for mastigote-enriched cultures, four replicates for coccoid-enriched cultures; Supplementary Table 1) and mapped the reads to the set of scaffolds from Aranda et al.^14^. Combined, a total of 2,324,324,062 uniquely mapping valid pairs of chromatin interactions was obtained. The Hi-C data were binned at 40-kb resolution and the interaction matrix was corrected for intrinsic experimental biases by balancing using the iterative correction method^29^. Scaffolds smaller than 40 kb were not included in the assembly process.

### Genome assembly: Smic1.0

We started assembly of the *S.* *microadriaticum* clade A genome with a set of scaffolds generated from Illumina HiSeq reads as described in Aranda et al.^14^. Combined, these scaffolds cover 808,242,489 bp of sequence data over 9,695 scaffolds. The scaffold N50 is 573.5 kb with a contig N50 of 34.9 kb (ref. ^14^).

We generated four Hi-C datasets for mastigote- and four for coccoid-enriched cultures of *S.* *microadriaticum* (D1, D3, D4, D7, see *Symbiodinium microadriaticum* culturing) in two biological replicates. The 16 datasets were pooled, yielding a total of 4,940,728,852 reads that were then used for Hi-C-assisted genome assembly. A schematic outline of the process of Hi-C-assisted genome assembly is shown in Extended Data Fig. 1, and described in detail in the Supplementary Methods.

### Genome assembly: Smic1.1N

Using the *S.* *microadriaticum* PacBio sequencing data, we generated a de novo genome assembly with Flye v.2.5 (ref. ^65^). This assembly was used to incorporate DNA sequences previously not found in chromosomes of Smic1.0, leading to the Smic1.1N assembly. This approach is described in more detail in the Supplementary Methods.

### Hi-C domain boundary detection

For the scaling plot for Hi-C domains, the positions of Hi-C domains were first defined by their boundaries using matrix2insulation script from cWorld (https://github.com/dekkerlab/cworld-dekker/blob/master/scripts/perl/matrix2insulation.pl) using all combined data matrix file binned at 10-kb resolution^34^. The insulation window size was 500 kb. This analysis produces an insulation profile along chromosomes (examples of insulation plots are shown in Fig. 1c). Local minima in insulation profiles indicate the positions of Hi-C domain boundaries, and the script produces a list of such boundaries and their strength^34,41^. To define a set of high confidence Hi-C domain boundaries, we selected boundaries with a boundary strength ≥0.2. Given that local minimum detection has an error of around ±1 bin, we manually corrected all boundaries calls (that is, shifting the positions of boundaries 1–2 bins) based on visual inspection of the Hi-C interaction map. The final list included 441 Hi-C domain boundaries (not including chromosomes 83–94, which are too small for insulation analysis with the settings described above). For Smic1.1, a total of 446 boundaries were found (not including chromosomes 83–94).

To check boundaries in contigs (less than 24 consecutive Ns in a 30-kb window around the boundary) from the above list, boundaries in subscaffolds were filtered and then filtered again with 10 kb upstream and 10 kb downstream adding to the 10-kb boundary (30 kb total) where this 30-kb region has less than 24 consecutive Ns. For Smic1.0, we have 65 such boundaries and for Smic1.1 we identified 241 boundaries located within contigs.

### *P*(*s*) calculation

*P*(*s*) plots were calculated in two ways. First, *P*(*s*) was calculated genome-wide using valid chromatin interaction pairs with the following script from the cooltools package with all default settings (https://github.com/mirnylab/cooltools).

For *P*(*s*) calculations for single chromosomes at the level of chromosomal Hi-C domains, we used Hi-C data binned and balanced at 50-kb resolution. Hi-C domains borders were calculated by insulation analysis (see above). The grid of domain borders defines a set of squares throughout the Hi-C interaction map. *P*(*s*) was calculated for each square by plotting the average of each diagonal of 50-kb bins in the square as a function of *s*. For all squares not centered at the main diagonal, the values of *P*(*s*) for the smallest and largest *s* were left out because they are calculated only for 1 bin and thus are noisy.

### Genome annotation and analysis

GC content calculation, genome annotation and GO enrichment analyses are described in the Supplementary Methods.

### Reporting Summary

Further information on research design is available in the Nature Research Reporting Summary linked to this article.

## Online content

Any methods, additional references, Nature Research reporting summaries, source data, extended data, supplementary information, acknowledgements, peer review information; details of author contributions and competing interests; and statements of data and code availability are available at 10.1038/s41588-021-00841-y.

## Supplementary information

Supplementary Information Supplementary Methods.

Reporting Summary

Peer Review Information

Supplementary Tables Supplementary Tables 1–3.

Supplemental Data File 1 Gene annotation for the Smic1.0 assembly.

Supplemental Data File 2 GFF3 file of Smic1.0 to be used with a genome browser.

Supplemental Data File 3 GFF3 file of Smic1.1N to be used with a genome browser.

Supplemental Data File 4 GO enrichment analysis (Biological Process) per chromosome and cluster (Smic1.0).

Supplemental Data File 5 GO enrichment analysis (Molecular Function) per chromosome and cluster (Smic1.0).

Supplemental Data File 6 GO enrichment analysis (Cellular Component) per chromosome and cluster (Smic1.0).

## Data Availability

All Hi-C and PacBio sequencing data and Smic1.0 and Smic1.1 genome sequences are available in GEO (accession number GSE152150). RNA-seq data used here are from Liew et al.^11^ and are available in NCBI Short Read Archive (SRA), BioProject PRJNA315758.

## Code availability

cMapping and distiller pipelines for Hi-C analysis are available at: https://github.com/dekkerlab/cMapping,https://github.com/mirnylab/distiller-nf. Hi-C-assisted genome assembly can be found at: https://github.com/dekkerlab/DNATriangulation. Tools for Hi-C data analysis can be found at: https://github.com/mirnylab/cooltools.
