# Supplementary material for: Genetic and spatial organization of the unusual chromosomes of the dinoflagellate Symbiodinium microadriaticum
Source: Nat Genet. 2021 Apr 29;53(5):618–29. doi: 10.1038/s41588-021-00841-y (PMC8110479; doi:10.1038/s41588-021-00841-y)
Supplement: Supplementary file 1 — Supplementary Methods. [file 41588_2021_841_MOESM1_ESM.pdf]

nature portfolio

https://doi.org/10.1038/s41588-021-00841-y

Supplementary information

Genetic and spatial organization of the unusual chromosomes of the dinoflagellate *Symbiodinium microadriaticum*

In the format provided by the
authors and unedited

Supplementary Methods

Hi-C protocol

Fixation

Hi-C was performed with cultures enriched in mastigotes or enriched in coccoid cells.

Mastigotes:

Mastigote-enriched cultures were obtained by collecting supernatants of cultures. Cells were fixed with 1% formaldehyde in seawater at RT for 10 min. Fixation was stopped by addition of glycine to a final concentration of 125 mM. Cells were incubated at RT for 5 min, followed by on ice >15 min. Cells were pelleted down and dissolved in seawater. Cell mix was aliquoted into tubes each containing 20 million cells. Cells were pelleted again and incubated on dry ice for more than 20 min then stored at -80C.

Coccoids:

After the removal of the supernatant of cultures (to remove mastigotes), remaining coccoid cells that were attached to the bottom of the flasks were fixed with 1% formaldehyde in 9 ml seawater at RT for 10 min. Fixation was stopped by addition of glycine to final concentration of 125 mM. Cells were incubated at RT for 5 min, followed by on ice >15 min. Cells were pelleted down and dissolved in seawater. Cell mix was aliquoted into tubes each containing 20 million cells. Cells were pelleted again and incubated on dry ice more than 20 min then stored at -80°C.

In some experiments Hi-C was performed with cells fixed with formaldehyde and Disuccinimidyl glutarate (DSG). Clone D4 coccoid-enriched cultures were fixed in 1% formaldehyde as described above. After stopping fixation by adding glycine to a final concentration of 125 mM, cells were scraped off the plates. The cells were washed twice in PBS and then resuspended in PBS containing 3 mM DSG. Cells were incubated at room temperature for 40 minutes with rotation. Fixation was stopped by addition of glycine to a final concentration of 400 mM. Cells were incubated for 5

minutes at room temperature, and then pelleted. Cells were washed twice in PBS, pelleted and flash frozen. Fixed cells were then stored at -80°C.

Cell lysis and restriction digestion

Hi-C was performed on 20 million cells per culture. Cells were resuspended in ~260 μl 1XNEBuffer 3.1 containing protease inhibitors (Thermo Scientific) and then split over 2 Covaris microTubes (Covaris, Part #520045). Cells were then sonicated. For coccoid cells the settings were: 90 seconds, Covaris M220 with the following parameters - peak power 75 watt, duty factor 23 and 200 cycles per burst. For mastigotes the settings were: 20 seconds, Covaris M220 with the following parameters - peak power 75 watt, duty factor 23 and 200 cycles per burst.

Each sample was then transferred to a microfuge tube and 1XNEBuffer 3.1 buffer was added to a total volume of 200 μl. Next, 10 μl 10% SDS was added to a final concentration of 0.5%, and cells were incubated at room temperature for 10 minutes. 23.6 μl 10% Triton X-100 was added to a final concentration of 1%, suspensions were gently mixed and then centrifuged at 3,000 *g* for 5 minutes. The supernatant was discarded, and each pellet was resuspended in 200 μl 1XNEBuffer 3.1. Samples were pooled, centrifuged again and pellets were resuspended in 490 μl 1XNEBuffer 3.1. Each sample was then split over 4 microfuge tubes (118 μl per tube), 8 μl DpnII (400 U, NEB, R0543M) was added to each and samples were incubated at 37°C overnight with rotation.

Biotin fill-in of DNA ends, DNA ligation and DNA purification

After overnight digestion, 354 μl 1XNEBuffer 2 was added to each sample. DNA ends were filled in with biotin-14-dATP by adding 60 μl of 1XNEBuffer 3 containing 0.25 mM dCTP, 0.25 mM dGTP, 0.25 mM dTTP, 0.25 mM biotin-14-dATP and 50 U Klenow. Samples were incubated at 23°C for 4 hours in a thermomixer and then placed on ice. DNA was ligated by adding 612 μl of ligation mix (final concentrations in reaction: 50 mM Tris-HCl (pH 7.6), 10 mM $MgCl_2$, 1 mM ATP, 1 mM DTT, 5% (w/v) polyethylene glycol-8000, 1% Triton X-10, 0.1mg/ml BSA) and 50 μl T4 DNA ligase (50 units)

followed by incubation at 16°C in a thermomixer for overnight. Next, 50 μl 10 mg/ml Proteinase K was added to each sample. Samples were then incubated at 65°C for 4 hours after which 50 μl 10 mg/ml Proteinase K was added again followed by incubation at 65°C overnight. The 4 samples were then pooled in one 15 ml conical tube and mixed with an equal volume phenol-chloroform (1:1). The sample was then transferred to a MaXtract™ tube and then centrifuged at 1,500 *g* for 5 minutes. The aqueous phase was transferred to a clear high-speed Beckman centrifuge tube, and DNA was precipitated by adding 1/10 volume 3M Sodium Acetate, pH 5.2 and 2.5 volumes ice cold 100% ethanol. Samples were incubated at -80°C for at least 60 minutes and then centrifuged at 18,000 *g* at 4°C for 20 minutes. The supernatant was removed, the pellet was dried and then resuspended in 800 μl EB buffer (10 mM Tris-Cl, pH 8.5) and split over two LoBind tubes. DNA was then purified on AMPure beads as follows: 2 volumes of AMPure mix was added followed by 10 minutes incubation at room temperature. Beads were reclaimed using a magnet, supernatant was removed followed by addition of 1 ml 80% ethanol. After 30 seconds of incubation the supernatant was again removed. Beads were washed once more by addition of 1 ml 80% ethanol and then beads were dried at room temperature for 5 minutes. Pellets were resuspended in 50 μl EB buffer and incubated at room temperature for 10 minutes. Beads were reclaimed with a magnet and the supernatant was transferred to a microfuge tube. RNA was removed by addition of 1 μl 10 mg/ml RNase A and incubation at 37°C for 15 minutes.

<u>Removal of dangling ends</u>

Biotin was removed from unligated ends by incubating samples (aliquots of 5 μg DNA, typically 10-15 μg per experiment) in 50 μl 1XNEBuffer containing 0.1 mg/ml BSA, 0.025 mM dATP, 0.025 mM dGTP and 15 U T4 DNA polymerase for 4 hours at 4°C. Reactions were then pooled in one LoBind tube, 2 volumes of AMPure mix were added and beads were reclaimed on a magnet. DNA was eluted in 130 μl water.

<u>Preparation of Hi-C libraries for Illumina sequencing</u>

DNA samples were transferred to Covaris microTubes, and sonicated for 3 minutes using a Covaris M220 with the following settings: peak power 50 watt, duty factor 20%,

200 cycles per burst. DNA ends were then repaired as follows: 120 µl DNA was mixed with 16 µl 10XNEB ligation buffer, 14 µl 2.5 mM dNTPs, 15 U T4 DNA polymerase, 50 U T4 polynucleotide kinase and 5 U Klenow DNA polymerase in a final volume of 161 µl. Samples were incubated at 20°C for 30 minutes. DNA was then purified by binding to QIAGEN MinElute columns (5 µg DNA per column), washing with 750 µl PE buffer, and DNA was then eluted twice with 17 µl TLE buffer. Next, A-tailing of the DNA molecules was performed by mixing 32 µl of DNA sample with 5 µl 10X NEBuffer 2, 10 µl 1 mM dATP and 15 U Klenow DNA polymerase (3'→5' exo-). Samples were incubated at 37°C for 30 minutes, followed by incubation at 65°C for 20 minutes. Samples were then placed on ice.

To purify biotin containing DNA fragments, TLE buffer was added DNA samples to make a final volume of 200 µl. Magnetic streptavidin beads (25 µl beads per 5 µg DNA) were washed twice in TWB (Tween Wash Buffer: 5 mM Tris-HCl pH8.0, 0.5 mM EDTA, 1 M NaCl, 0.05% Tween), resuspended in 200 µl 2X Binding Buffer (BB) and added to 200 µl DNA solution. The mixture was incubated at room temperature for 15 minutes with rotation. The beads were reclaimed on a magnet and the supernatant was discarded. Beads were resuspended in 200 µl 1X BB beads and were reclaimed again on a magnet and the supernatant was discarded. Beads were then resuspended in 100 µl 1XT4 DNA ligation buffer (Invitrogen), and transferred to a new tube. The beads were reclaimed on a magnet again, the supernatant was discarded and then resuspended in 40.75 µl 1XT4 DNA ligation buffer (Invitrogen).

To prepare DNA (bound to the streptavidin beads) for Illumina sequencing the DNA sample was mixed with 4 µl Illumina paired end adapters (TruSeq Nano DNA Sample Prep Kit, FC-121-4001), 2.25 µl 5X T4 DNA ligation buffer (Invitrogen) and 3 µl T4 DNA ligase (Invitrogen). All reactions were performed in LoBind tubes. Mixtures were incubated at room temperature for 2 hours. Beads were then reclaimed on a magnet and beads were washed in several steps as follows: first two washes with 300 µl TWB, third wash with 200 µl 1X BB, fourth wash with 200 µl 1X NEBuffer 2 and finally with 50

μl 1X NEBuffer 2. After the last wash the beads were resuspended in 20 μl 1X NEBuffer 2 and then transferred to a new microfuge tube.

DNA was then amplified according to the TruSeq Nano DNA Sample Prep Kit, FC-121-4001 for 6-9 cycles and amplified DNA was purified using AMPure as follows: DNA solution was mixed with 1.1X volume of AMPure XP and incubated at room temperature for 10 minutes. Beads were reclaimed with a magnet and the supernatant was discarded. The beads were then twice washed with 500 μl fresh 80% ethanol. Beads were air-dried for 5 minutes and then resuspended in 30 μl EB and incubated at room temperature for 10 minutes. Beads were reclaimed again and the supernatant was transferred to a new microfuge tube. DNA concentration was then determined by gel analysis.

DNA sequencing, Hi-C read mapping and analysis

Hi-C libraries were analyzed by 2X50 bp paired-end sequencing on a HiSeq4000 instrument. Reads were mapped using the cMapping pipeline (https://github.com/dekkerlab/cMapping) or distiller pipeline (https://github.com/mirnylab/distiller-nf). Reads were initially mapped to *S. microadriaticum* scaffolds from Aranda et al. [1] to facilitate Hi-C assisted genome assembly, and finally to the assembled genome version Smic1.0.

PacBio library preparation and sequencing

Genomic DNA was extracted from coccoid-enriched and mastigote-enriched cultures of clone D7 (growing in the presence of antibiotics, see above) using the QIAGEN DNeasy Plant Mini Kits (QIAGEN, Cat# 69104). The cells were ground to a fine powder under liquid nitrogen using a mortar and pestle. Once cell disruption was complete, DNA extraction was performed following the manufacturer's protocol. DNA from mastigote-enriched cultures was extracted using QIAshredder Mini spin columns while DNA from coccoid enriched cultures was extracted both with and without QIAshredder Mini spin columns.

A first set of DNA libraries were sequencing on a PacBio RS II, and later two libraries were sequenced on a PacBio Sequel I instrument (see Supplemental Table S3). Initial quality control analysis was performed on all samples using Q-Bit, NanoVue, Advanced Analytics-based DNA Fragment Analysis. For initial analysis, material used in libraries analyzed on the PacBio RS II instrument was unsheared as quality control analysis revealed it was already quite fragmented. All samples underwent cleanup steps prior to library construction: Two 0.5X AmpPure bead washes.

In preparation of sequencing DNA on the PacBio Sequel I, DNA was needle sheared: A 1 mL Luer-Lok syringe with 26G 1.5” blunt needles was used for shearing: Sample mD7 (mastigote DNA) was passed 10 passes through the needle, sample cD7 (coccoid DNA), which initially had a smaller starting size and shoulder on the initial quality control, was passed only 5 passes through the needle. Sheared material was assessed using a high-sensitivity DNA Fragment Analyzer assay.

All libraries were of the long-insert genomic DNA type, all constructed using the PB Express 2.0 Kit according to the manufacturer’s instructions. An additional cleanup step was performed following the completion of library construction. Validation quality control analysis performed on all finished libraries included Q-Bit, NanoVue, Advanced Analytics-based DNA Fragment Analysis.

Libraries that were analyzed on the RS II instrument used one SMRTCell with a 10-hour data collection time; libraries analyzed on a Sequel I used one (1M) SMRTCell with a 20-hour data collection time. Read-of Insert (ROI)/ CCS analysis was performed using SMRTLink v.6 or SMRTLInk v.7.

Genome assembly

Hi-C data was mapped to the set of scaffolds using the standard cMapping pipeline [[2] and https://github.com/dekkerlab/cMapping]. Out of a total of 4,940,728,852 Hi-C paired-end reads, for 2,324,324,062, i.e. 47.04%, both ends uniquely mapped to

scaffolds. This is comparable to Hi-C data for the human genome where the fraction of uniquely mapping paired end reads is typically around 60%, indicating that the set of scaffolds represents a large majority of the *S. microadriaticum* genome. Of the set of uniquely mapped paired-end reads 1,379,534,687 (59.35%) represented interactions between scaffolds and 944,789,375 (40.65%) represented interactions within scaffolds. Hi-C data was then binned at 40 kb resolution. The final assembly Smic1.0 has 94 chromosomes covering 624,473,910 bp. In addition, for each chromosome we identified a set of sub-scaffolds that are present as high copy number sequences which made correct positioning of them along the chromosomes difficult. Combined these high copy number sub-scaffolds cover 183,768,579 bp.

<u>Removal of small scaffolds and bins at ends of scaffolds that are smaller than 40 Kb</u>
Scaffolds smaller than 40 kb were removed due to the relatively low read coverage in Hi-C datasets. Low read coverage will affect normalizing the Hi-C interaction matrix after balancing. Out of 9,695 scaffolds, 7,671 scaffolds smaller than 40 Kb were removed. After binning Hi-C data at 40 kb resolution, each scaffold larger than 40 kb will have a last bin at their 3' end that is smaller than 40 kb. Those so-called "hanging bins" were also removed. Combined 9,695 bins were removed covering ~70 Mb. The remaining interaction matrix contained 18,468x18,468 bins of 40 kb covering 738,720,000 bp. The Hi-C interaction matrix was then normalized for technical biases by balancing using the conventional Iterative Correction and Eigenvector decomposition (ICE) method [3].

<u>Karyotyping</u>
Loci (bins) interact more frequently with other loci located along the same chromosome (in cis) than with loci located on other chromosomes (in trans). This feature can be leveraged to identify sets of bins that all interact with each other more frequently than with others and thus are present on the same chromosome. We refer to this step as karyotyping and it involves converting the Hi-C interaction matrix into a genomic distance matrix followed by bootstrapped clustering of bins based on the distances between pairs of 40 kb bins. We used the algorithm and code as described in Kaplan et

al [4]. We run the clustering 100 times randomly picking 90% of the data in each iteration and then estimated the number of clusters by identifying the largest average distance step in the hierarchical trees which occurred at 82 clusters. We assume that each of these clusters represents a set of loci (40 kb bins) located on the same chromosome.

Removal of erroneous bins

We noticed the presence of 40 kb bins that clustered with a set of other bins but also displayed high interactions with bins within other clusters, indicating they contained sequences present on two different chromosomes. These bins may contain "misjoins" where the original scaffolds contained incorrectly joined sequences from two chromosomes. Bins were assumed to be erroneous when the sum of their interactions with bins outside their cluster was above 300 counts. These bins were removed. This led to the removal of 5,239 bins covering 209,560,000 bp. The remaining interaction matrix consisted of 13,229x13,229 bins of 40 kb covering 529,160,000 bp.

Creation of sub-scaffolds

Bins that were clustered together, did not contain misjoins and that were adjacent within the same original scaffold were merged to form "sub-scaffolds". Sub-scaffolds are parts of original scaffolds where they were linked to other sub-scaffolds by misjoins. Sub-scaffolds are high confidence scaffolds: they were originally assembled using short reads by Aranda et al., their 40 kb bins were found to be located on the same chromosome (cluster) by Hi-C, and they do not contain misjoins using the threshold described above. We created 3,202 sub-scaffolds again performed karyotyping to group sub-scaffolds located along the same chromosome and again identified 88 clusters. 10 subs-scaffolds (400 kb of sequence) were removed from the matrix at this step by DNA triangulation filters. Hence we assembled 3,192 sub-scaffolds that combined cover 528,760,000 bp.

De novo scaffolding

Next we set out to order sub-scaffolds along chromosomes. To this end we mapped the pooled Hi-C data to individual sub-scaffolds and binned the Hi-C data so that each bin

contains a full length single sub-scaffold. The interaction map was then balanced using ICE [3] to create a normalized interaction matrix of 3,192x3,192 bins. We then used Hi-C interaction frequencies between sub-scaffolds within each cluster to order them along chromosomes in a process referred to as "scaffolding" as described by Kaplan [4]. Scaffolding is based on the fact that Hi-C interaction frequencies decay with genomic distance. We modified the previously published scaffolding algorithm [4] and used a probabilistic model that assumes that the distance dependent decay follows a power law to find sets of likely sub-scaffold positions for each chromosome. The solution space is not concave and individual solutions may represent local minima. Therefore, we repeated the optimization with 10 different starting points and through 1000 iterations with an optimization algorithm L-BFGS (Limited-memory Broyden–Fletcher–Goldfarb–Shanno algorithm) we identified the best solution which was reported as the ordering of sub-scaffolds within each cluster. Next we created a new interaction matrix where each chromosome was composed of the newly ordered sub-scaffolds split in their original 40 kb bins. The new chromatin interaction matrix has 13,219x13,219 bins of 40 kb = 528,760,000 bp covering 88 chromosomes (Matrix expansion step).

<u>Manual correction of clustering, ordering and orientation of sub-scaffolds</u>

Visual inspection of the chromatin interaction matrix revealed errors. First, some 40 kb bins were assigned to the incorrect cluster. We manually assigned such bins to the cluster it interacts most strongly with. Second, errors in ordering of sub-scaffolds along chromosomes were visible by sharp breaks and checkered appearance of the interaction maps characteristic for inversions and re-arrangements [5]. These errors were manually corrected to create chromatin interaction maps with smooth distance dependent decay in Hi-C interactions. Third, all sub-scaffolds were oriented according to the original scaffold sequences and thus about half were expected to be in the incorrect orientation. For sub-scaffolds represented by multiple 40 kb bins the orientation could be inferred by examining interactions with their flanking sub-scaffolds. We manually oriented each of the >40 kb sub-scaffolds so that their interactions with flanking sub-scaffolds followed a smooth distance dependent decay. Sub-scaffolds composed of a single 40 kb bin are oriented below.

For all manual corrections of the assembly, at this stage and below, we attempted to maintain the continuity of bins within sub-scaffolds and only moved bins away from other bins of the same sub-scaffold when the Hi-C interaction pattern was very obviously incorrect.

Adding back previously removed bins

In the beginning of the assembly process we had removed 40 kb bins that displayed interaction counts >300 with clusters to which they were not assigned (“erroneous bins”, above). We noted that some of these bins were consecutive “chunks” of 80 or more kb within the original scaffolds. We reasoned that many of such large chunks of multiple 40 kb bins would not contain misjoins and could be placed in the current assembly. To this end we created a new fasta file composed of the 88 clusters assembled above, as well as the sequence of all erroneous chunks of 80 kb or larger (479 chunks). We mapped the Hi-C data to this sequence and balanced the interaction matrix using ICE [3]. We then applied the karyotyping procedure to the 2,543x2,543 40 kb bin interaction matrix containing the chunks and identified 77 clusters. Each of these 77 clusters was then manually assigned to one the 88 clusters assembled above, and inserted in their appropriate locations and orientation to accommodate smooth distance dependent decay of their interactions with the other sub-scaffolds present. In addition, we identified 3 clusters of chunks that did not interact frequently with any of the 88 clusters assembled above indicating that these represent 3 additional chromosomes. We added these three clusters as separate chromosomes so that the total number of clusters / chromosomes at this stage of the assembly was 91. In total we added 2,543 bins of 40 kb (101,720,000 bp) back into the assembly that now covers 630,480,000 bp. We also were able to place 4 bins of 40 kb that had been removed by triangulation filters above so that the assembly totals 630,640,000 bp.

After adding back the erroneous chunks we re-evaluated the cluster assignment of each bin. We identified 180 bins of 40 kb that were clearly mis-assigned and manually placed them within the correct cluster they interacted most frequently with.

Orienting sub-scaffolds composed of single 40 kb bins

The assembly contains 374 40 kb bins that either represent a single full sub-scaffold, or that had been manually separated from other 40 kb bins from the same sub-scaffold to ensure smooth distance dependent decay in Hi-C interactions. In order to orient these singletons we first mapped the Hi-C data to the current assembly and binned the data at 8 kb resolution so that interactions between the left end and the right end of these 40 kb sub-scaffold with flanking sequences could be manually evaluated and the sub-scaffolds could be oriented to ensure smooth distance dependent decay.

Adding back hanging bins

As outlined above during the first steps of the assembly process the final 3' end bins of the original scaffolds from Aranda et al. [1] that were less than 40 kb ("hanging bins) were removed. In total 9,695 hanging bins covering 69,522,489 bp were left out of the assembly. At this step these hanging bins could be placed back in the appropriate position and orientation if the adjacent bin from the original scaffold was present in the assembly. In total 1,541 hanging bins covering 30,548,811 bp could be placed back into the assembly that now makes up 661,188,811 bp. A new fasta files was created, and the pooled Hi-C data was mapped to this genome and interaction data was now binned at 1 kb resolution.

Manual curation of genome assembly at 1 kb resolution

In a final refinement of the assembly we assessed Hi-C interaction frequencies at 1 kb resolution. We removed 1 kb bins when the sum of all their interaction frequencies with other bins along the same chromosome was below 60% of the most frequent (Max) sum of all intra-chromosomal interactions for all 1 kb bins along the same chromosome (see figure below) . Such 1 kb bins display relatively high interaction frequencies with one or more other chromosomes and may contain sequences that are incorrectly included in the scaffolds generated by Aranda et al. [1].

At this step 32,400,317 bp were removed from the assembly. In addition, two clusters were deemed to be composed of 2 and 3 clusters respectively and split accordingly, adding 3 new clusters (chromosomes) to the assembly. At this stage the assembly contains 94 chromosomes covering 628,788,494 bp.

Final manual removal of erroneous bins & correction of bin orientations

The last step of the assembly involved visual inspection of Hi-C interaction maps of all 94 chromosomes at 1 kb resolution. Any 1 kb bin for which the Hi-C distance dependent decay pattern appeared erroneous was removed. In total 4,314,584 bp were removed.

Final assembly Smic1.0

The final assembly contains 94 chromosomes covering 624,473,910 bp. We arranged chromosomes in order of decreasing size of the clusters to obtain assembly version Smic1.0.

Cluster 95: Non-assembled high-copy sequences

All sequences from the original set of scaffolds assembled by Aranda et al. [1] that could not be placed on chromosomes 1-94 (in total 183,768,579 bp) were concatenated to form "cluster 95".

Copy number analysis

Hi-C raw single end read coverage analysis of chromosomes 1-94 and cluster 95 revealed that loci along the 94 assembled chromosomes displayed very similar copy number. In contrast, sequences in cluster 95 displayed higher copy numbers (average 11X higher than sequences on chromosomes 1-94). This indicates that the (sub-) scaffolds that make up cluster 95 are present at high copy number and this may explain in part the fact that these could not be placed consistently or with confidence at defined positions along chromosome 1-94.

Assigning high copy sub-scaffolds to chromosomes 1-94

To further investigate sub-scaffolds that make up cluster 95, we re-mapped the Hi-C data using the distiller pipeline (https://github.com/mirnylab/distiller-nf) to a new fasta file which includes chromosomes 1-94 and all remaining sub-scaffolds which were left out of the assembly as separate entries to the fasta file (31,552 sub-scaffolds). We then binned and Iced the data at 1Mb resolution. Note that because sub-scaffolds are much smaller than 1 Mb, each will simply contain a single full length sub-scaffold. Next, we calculated the average size-normalized Hi-C interaction frequency between each bin and each of chromosomes 1 through 94 to identify the chromosome each sub-scaffold bin interacts with mostly. In several cases we could not assign the chromosome a given sub-scaffold interacts mostly with (e.g. due to zeros in the contact map). In those cases we assigned such sub-scaffolds to the cluster to which the previous sub-scaffold from the same original Illumina-based scaffold was assigned. Sub-scaffolds that all interact with the same chromosome were then concatenated to form a single set. Sub-scaffolds that could not be assigned to any specific chromosomes were concatenated to form a separate set (number 95). Finally, a new fasta file was created that contained chromosomes 1-94, followed by 94 sets of sub-scaffolds that interact with chromosomes 1-94 respectively, followed by a final set (number 95) of sub-scaffolds that could not be assigned to any chromosome. This fasta file includes all the sequence i.e. 808,242,489 bp that made up the set of Illumina-based scaffolds generated by Aranda et al. [1].

PacBio assembly and the generation of Smic1.1N

Using the *S. microadriaticum* PacBio sequencing data we generated a *de novo* genome assembly with Flye v.2.5 [6]. Since PacBio reads are much longer than Illumina reads, they are able to span through mid-length repetitive sequences, enabling them to be integrated into contigs. This assembly was used to incorporate DNA sequences previously not found in chromosomes of Smic1.0, leading to the Smic1.1N assembly. To generate Smic1.1N, we first added 100 Ns between each contig that was joined through Hi-C data in Smic1.0, leading to Smic1.0N. Then we mapped PacBio derived contigs, from the Flye assembly, to Smic1.0N with Minimap2 [7]. Knowing the coordinates of the mapped PacBio contigs, we replaced the Illumina derived sequences (Smic1.0N) with

PacBio sequence (Flye) at the mapping locations. If a PacBio contig spanned through a chromosome region, yet it contained additional sequence than that found at the corresponding chromosomal region, the full PacBio region including the additional sequence was integrated into the chromosome between the starting and ending consecutive mapping coordinates between the chromosome and PacBio contig. When the PacBio derived contigs mapped to the negative strand, the reverse complement of the PacBio derived contig was incorporated. In case two contigs mapped to the same region of a chromosome, the contig covering the longest region was incorporated. The resulting genome was then polished with Pilon v.1.23 [8] using Illumina reads and ran under default parameters, resulting in Smic1.1N.

P(*s*) calculations and estimation of gap sizes between Hi-C domains

When we assume that Hi-C domains are the result of gaps in the assembly, we can estimate the size of the gaps by calculating what the genomic distance should be between two loci immediately adjacent of a Hi-C domain boundary given the observed interaction frequency between them. To estimate the sizes of gaps for a few chromosomes we calculated *P*(*s*) plots for all on-diagonal Hi-C domains for those chromosomes and for the squares in the Hi-C interaction maps that are positioned immediately off-diagonal and represent interactions between adjacent Hi-C domains. We then estimated the sizes of the gaps at the boundaries between Hi-C domains by determining how much the *P*(*s*) plots of the off-diagonal squares needed to be shifted along the x-axis to make them smoothly overlap with the *P*(*s*) plots of the on-diagonal domains. Interestingly, application of gaps estimated in this manner make *P*(*s*) plots of all squares of the Hi-C maps overlap more smoothly. This included *P*(*s*) plots for squares of the Hi-C maps that correspond to interactions between Hi-C domains that are separated by more than 1 boundary/gap.

Genome annotation and analysis

Identification and masking of repetitive elements

Repetitive elements in the Hi-C scaffolded genome were identified and masked with RepeatMasker (Smit A, Hubley R, Green P. *RepeatMasker Open-4.0* 2013-2015 [Available from: http://www.repeatmasker.org) using the *de novo* repeat library for *S. microadriaticum* generated by Aranda et al. [1]. This resulted in masking 26.45 % of the genome, of which the most abundant repetitive elements were LINES, DNA transposons, simple repeats, and unclassified. More than 50 % of the repetitive elements were LINEs, comprising 13.36 % of the genome. To observe the distribution of repetitive elements along chromosomes, we measured the abundance of the most prominent repetitive elements using 100 kb non-overlapping windows.

Genome annotation, enrichment analyses, and gene expression

As the Hi-C scaffolded genome is based on the previous *S. microadriaticum* assembly [1], the annotation of the Hi-C scaffolded genome consisted of remapping the annotation of the previously generated genome by Aranda et al. [1] to the Hi-C scaffolded genome with Minimap2 [7]. 48,715 out of 49,109 genes were mapped from the original assembly to the new Hi-C scaffolded genome. GO enrichment analysis was done at a chromosome level with topGO (version 2.37.0; Bioconductor package Alexa A, Rahnenfuhrer J (2020). *topGO: Enrichment Analysis for Gene Ontology*.) and evaluated with weight01 Fisher statistic at a 0.05 p-value threshold. KEGG orthology was assigned with BlastKOALA (28). To assess gene expression across different regions of the chromosomes, RNASeq reads previously generated by Aranda et al [1], were mapped to the Hi-C scaffolded genome using HiSAT2 (v. 2.1.0) [9].

Gene distribution and orientation analyses

Gene distribution along chromosomes was measured as the number of genes found in 100 kb non-overlapping windows. The distribution of genes in blocks of co-oriented genes was measured by counting the number of consecutive genes found on the same strand (plus or minus) until the next neighboring gene appeared on the opposite strand. Gene orientation changes were measured as the number of times neighboring genes appeared on opposite strands within a 10 gene sliding window and compared to orientation changes assuming an equal probability and independent occurrence of genes at either strand using a binomial distribution.

GC content

GC content was measured only in defined regions where at least 50 % of the bases were [A,C,G,T], meaning that regions containing 50 % Ns were not used for the analysis. GC content along chromosomes was measured in 10 (for plotting) and 100 (for correlation analysis) kb non overlapping windows. GC content surrounding insulation boundaries was measured in 100 bp sliding windows across 70 kb regions that included 30 kb upstream and 30 kb downstream of the 10 kb insulation boundaries.

Telomeres analyses

Analyses of telomeric regions were done using 2.5 Mb from each telomeric end, meaning that chromosomes smaller than 5 Mb were not included in the analyses. This resulted in 69 chromosomes utilized for the analyses. Gene number, gene directionality, and LINEs number were measured at window sizes of 100 kb, whereas GC content was measured at 10 kb windows. For every plot of each analysis, a polynomial of the fourth order fit was derived together with the respective coefficient of determination (R^2).

Correlations at a chromosome level

Correlations between Gene number, GC content, RNASeq, and repetitive elements: LINEs, DNA transposons, Simple repeats, and Unclassified repeats, were performed using values from 100 kb non-overlapping windows. Correlations were performed in R with the corrplot package (v. 0.84) using a Pearson's correlation and corrected for multiple testing with Benjamini-Hochberg procedure at a 0.05 p-value threshold.

References

1. Aranda, M. *et al.* Genomes of coral dinoflagellate symbionts highlight evolutionary adaptations conducive to a symbiotic lifestyle. *Sci Rep* **6**, 39734 (2016).
2. Lajoie, B.R., Dekker, J. & Kaplan, N. The Hitchhiker's guide to Hi-C analysis: Practical guidelines. *Methods* **72**, 65-75 (2015).

3. Imakaev, M. *et al.* Iterative correction of Hi-C data reveals hallmarks of chromosome organization. *Nature Methods* **9**, 999-1003 (2012).
4. Kaplan, N. & Dekker, J. High-throughput genome scaffolding from in vivo DNA interaction frequency. *Nat Biotechnol.* **31**, 1143-1147 (2013).
5. Dixon, J.R. *et al.* Integrative detection and analysis of structural variation in cancer genomes. *Nat Genet* **50**, 1388-1398 (2018).
6. Kolmogorov, M., Yuan, J., Lin, Y. & Pevzner, P.A. Assembly of long, error-prone reads using repeat graphs. *Nat Biotechnol* **37**, 540-546 (2019).
7. Li, H. Minimap2: pairwise alignment for nucleotide sequences. *Bioinformatics* **34**, 3094-3100 (2018).
8. Walker, B.J. *et al.* Pilon: an integrated tool for comprehensive microbial variant detection and genome assembly improvement. *PLoS One* **9**, e112963 (2014).
9. Kim, D., Paggi, J.M., Park, C., Bennett, C. & Salzberg, S.L. Graph-based genome alignment and genotyping with HISAT2 and HISAT-genotype. *Nat Biotechnol* **37**, 907-915 (2019).
