# Supplementary material for: Genetic and spatial organization of the unusual chromosomes of the dinoflagellate Symbiodinium microadriaticum
Source: Nat Genet. 2021 Apr 29;53(5):618–29. doi: 10.1038/s41588-021-00841-y (PMC8110479; doi:10.1038/s41588-021-00841-y)
Supplement: Supplementary file 3 — Peer Review Information [file 41588_2021_841_MOESM3_ESM.pdf]

natureresearch

Peer Review Information

Journal: Nature Genetics
Manuscript Title: Genetic and spatial organization of the unusual chromosomes of the dinoflagellate Symbiodinium microadriaticum
Corresponding author name(s): Dr. Job Dekker

Reviewer Comments & Decisions:

Decision Letter, initial version:

5th Aug 2020

Dear Job,

Your Article, "Chromosome-scale assembly of the coral endosymbiont Symbiodinium microadriaticum genome provides insight into the unique biology of dinoflagellate chromosomes" has now been seen by 3 referees. You will see from their comments below that while they find your work of interest, some important points are raised. We are interested in the possibility of publishing your study in Nature Genetics, but would like to consider your response to these concerns in the form of a revised manuscript before we make a final decision on publication.

Reviewer #1 is broadly positive about the manuscript and has some suggestions for improvement, mainly textual. It should straightforward to address their comments.
Reviewer #2 thinks the work is well done technically, overall. Their comments are mainly about data interpretation and manuscript structure.
Reviewer #3 finds the work quite interesting. The two main requests are about improving the genome assembly, which is an important point, and about carefully analyzing chromosome structure throughout the cell cycle. The reviewer also suggests experimentally testing whether transcription drives 3D nuclear organization, but this is not essential.

We therefore invite you to revise your manuscript taking into account all reviewer comments. Please highlight all changes in the manuscript text file. At this stage we will need you to upload a copy of the

manuscript in MS Word .docx or similar editable format.

We are committed to providing a fair and constructive peer-review process. Do not hesitate to contact me if there are specific requests from the reviewers that you believe are technically impossible or unlikely to yield a meaningful outcome.

When revising your manuscript:

*1) Include a “Response to referees” document detailing, point-by-point, how you addressed each referee comment. If no action was taken to address a point, you must provide a compelling argument. This response will be sent back to the referees along with the revised manuscript.

*2) If you have not done so already please begin to revise your manuscript so that it conforms to our Article format instructions, available
here.
Refer also to any guidelines provided in this letter.

*3) Include a revised version of any required Reporting Summary:
https://www.nature.com/documents/nr-reporting-summary.pdf
It will be available to referees (and, potentially, statisticians) to aid in their evaluation if the manuscript goes back for peer review.
A revised checklist is essential for re-review of the paper.

Please be aware of our guidelines on digital image standards.

Please use the link below to submit your revised manuscript and related files:

https://mts-ng.nature.com/cgi-bin/main.plex?el=A5G3BQG2A6ZVm4J5A9ftdea6FsEukV1h2zus1fnDgZ

Note: This URL links to your confidential home page and associated information about manuscripts you may have submitted, or that you are reviewing for us. If you wish to forward this email to co-authors, please delete the link to your homepage.

Please do not hesitate to contact me if you have any questions or would like to discuss these revisions further.

Nature Genetics is committed to improving transparency in authorship. As part of our efforts in this direction, we are now requesting that all authors identified as ‘corresponding author’ on published

papers create and link their Open Researcher and Contributor Identifier (ORCID) with their account on the Manuscript Tracking System (MTS), prior to acceptance. ORCID helps the scientific community achieve unambiguous attribution of all scholarly contributions. You can create and link your ORCID from the home page of the MTS by clicking on 'Modify my Springer Nature account'. For more information please visit please visit www.springernature.com/orcid.

We look forward to seeing the revised manuscript and thank you for the opportunity to review your work.

Sincerely,

Tiago

Tiago Faial, PhD
Senior Editor
Nature Genetics
https://orcid.org/0000-0003-0864-1200

Referee expertise:

Referee #1: protist biology

Referee #2: 3D genomics

Referee #3: 3D genomics

Reviewers' Comments:

Reviewer #1:
Remarks to the Author:
In this manuscript, Nand et al. use a combination of DNA sequencing methodologies to generate a chromosome-scale genome assembly for the dinoflagellate Symbiodinium microadriaticum, which they

use to investigate three-dimensional genome architecture and its relation to genomic features. This represents a major feat that not only provides an invaluable resource for studying dinoflagellate genomics, but new insights into the biology of dinoflagellate chromosomes. Despite its importance, progress in understanding dinoflagellate chromatin biology has slowed, and this paper exemplifies how interdisciplinary approaches can move the field forwards.

In general, I found the paper to be well written, the results robust and thought-provoking. I have to note that I am not an expert in Hi-C, but the methods appear robust and echo the highly regarded work of the labs involved.

In general, my criticisms centre on making the manuscript more interpretable for a wider audience (particularly those not familiar with Hi-C and dinoflagellates) and integrating additional dinoflagellate concepts and literature. I also had some concerns about the gene ontology analysis. These should all be addressable through a few minor revisions to the text and analyses.

I feel the manuscript could benefit by better integrating concepts in dinoflagellate chromatin biology and genomics with the Hi-C methodology. The introduction needs more information on dinoflagellate chromosome structure and Hi-C (which chromatin biologists and protistologists may be less familiar with, respectively). We know a reasonable amount about the morphology of dinoflagellate chromosomes from microscopy studies (e.g., their linear/rod shaped conformation, the presence of banding and ridges, and the existence of loops which may be transcriptionally active) and replacement of bulk nucleosomes by DVNP and (in core dinoflagellates) HLPs, should be noted here. This would help place the results in context.

It would also be interesting during the discussion to talk more about current hypotheses of dinoflagellate chromosome structure (as was briefly done – lines 674-679) and discuss how they align with the Hi-C data. Which parts of these hypotheses agree with the Hi-C data? Which are refuted? What kind of Hi-C data would you expect from dinoflagellate chromosomes? What are the limits of the Hi-C data? Overall, I think this manuscript would be significantly more accessible if it included a detailed summary diagram depicting what this study has revealed about dinoflagellate chromosome structure and function.

Other comments:

Line 1: Is S. microadriaticum a coral endosymbiont? I only know it from jellyfish (e.g., Cassiopeia).

Line 26: See Line 1.Line 50: “enables” should be “enable”

Lines 71-76: This makes it sounds like dinoflagellates are neither eukaryotes nor prokaryotes (hearkens back to the incorrect idea that dinoflaglellates are ‘mesoeukaryotes’). This should be revised to avoid confusion.

Line 86: “or” should be “and”

Line 130-135: Can you add average or median chromosome sizes (or sizes of the assembled chromosome clusters)?

Line 174-175: This could use slightly more clarification. Why does copy number result in more inter-chromosomal interactions? Is it because at a small frequency each genomic region interacts with other chromosomes and when you collapse a multicopy region into a single bin, the small number of

interactions sums to be noticeable?
Line 177: Where in the chromosomes are these regions? Is it consistent between chromosomes? Given that each chromosome has one of these high copy regions, could they have functional significance (e.g., could they act as centromeres?)?
Line 186-195: This paragraph should be revised as it is unclear whether this is an introduction to interpreting Hi-C heatmaps generally or an interpretation of the data itself. At first I thought it was about the data, but then I saw the citation and no figure references and thought it was an introduction, and then it concluded somewhat abruptly with the comment on coccoid vs mastigote cells.
Line 202-203: Does “on most chromosomes” refer to “on canonical chromosomes”?
Figure 1B: The interactions between clusters 1-94 and other chromosomes, besides their main interacting chromosome, are very hard to see which makes it a bit confusing when reading the results. This could be a resolution issue.
Figure 1C: I think the edges of the plots should be labeled (e.g., chromosome vs chromosome and chromosome vs cluster)
Line 219-241: I understand the interpretation that the third regime in the P(s) implies a linear chromosome but I think it’s worth elaborating on the concept of pseudolayers. Do pseudolayers refer to the fact that within the rod, the genome is not significantly mixed along the vertical axis of the chromosome? If each locus interacts with regions 3Mbp upstream and downstream, it is unclear to me what the size of a pseudolayer refers to as it seems like the interactions should be a continuum across the chromosome. Could the size of regime II reflect other features of the chromosome such as its width?
Line310: Can you show the trend in GC for all chromosomes or averaged across chromosomes?
Line 318: Remove ‘and’
Line 341: “allows to elucidate” should be “allowed us to elucidate”
Line 343: “particular” should be “particularly”
Line 343: “have” should be “have been”
Line 356-357: Consider rewording as “resulting in a moderate yet significant positive correlation between gene density and gene expression (0.33 R2, 4.2e-157 p-value)
Line 359: Figure 4H is cited before other panels in Figure 4. It should be revised to keep things in order.
Line 359-360: Could the correlation between gene density and GC reflect GC codon bias in dinoflagellates (e.g., see Williams et al. 2017. Mar Drugs. 15(5): 125). Also isn’t it intuitive that more RNA-seq reads should map to a region if there are more genes in it? It would make sense to also look at expression level across the chromosome (i.e., where are the highest expressed genes and what genomic features do they correlate with?).
Figure 4A: Add genomic reads (e.g., like in Figure 1B) to these plots to show that increases in transcription are not a reflection of repeats and high copy number.
Figure 4H: The figure is missing negative values on the legend.
Line 399: Except for some eukaryotes, like kinetoplastids, where there are large co-oriented gene arrays.
Line 414: Why is it surprising?
Line 467-469: It is unclear what Figure 5B is showing and what the analysis indicates. Please elaborate

and add labels to the plot.
Line 488: “converges” should be “converge”
Line 524-573: Given the nature of the tandem repeats I find the gene ontology analysis looking for functional enrichment in chromosomes somewhat problematic. My main concern is that a large tandem array of a single gene could make it appear that a chromosome is enriched for a certain process. For example, chromosome four is apparently enriched for photosynthesis related genes but to what extent is this just a result of having six duplicated copies of ATP synthase subunit C? In other words, a chromosome may have lots of photosynthesis genes but only a marginal proportion of the photosynthesis pathway. Was this accounted for in the analysis (it was unclear from the methods)? If not, it would be worth trying to normalize for this. Perhaps this could be done by assigning genes to KEGG terms and looking for enrichment of pathway components (e.g., if there are X photosynthesis KOGs in the genome, can you reject the hypothesis that they are distributed randomly?). Another approach could be to collapse recent paralogs and duplications into single data points based on BLAST E-values or percent identity.
Line 524-525: More analysis of the tandem gene arrays would be worthwhile. For example, do the genes within an array tend to be related to a certain function? What about within chromosomes domains? Are genes within an array more closely related (e.g., recent paralogues) or are they more like operons, containing sets of functionally related genes? Based on line 527-529, it seems some of these questions may have been looked at but it was unclear where the data and results were.
Line 604-605: “of the unique clade of dinoflagellates,” should be “of the dinoflagellates.”
Line 610-615: Are the interactions between activating and inactivating domains in other eukaryotes dependent on the more homogenous nature/flexibility of interphase chromosomes? Since dinoflagellate chromosomes are permanently condensed, is it expected that there wouldn’t be these interactions? Interesting to think about what functional consequences or coping mechanisms there may be without these interactions.
Line 640-642: What about in Trypanosomes where there are large arrays of co-oriented genes? Has any correlation with domain structure been observed there? (See Muller et al. 2018. Nature 563:121-125)
Line 644-646: This is speculation, but given the similarities between genome conformation in Caulobacter and S. microadriaticum, it is interesting to wonder whether domains are in part defined by HLPs which dinoflagellates derived from bacteria through horizontal gene transfer. The acquisition of HLPs actually correlates with the origin of liquid crystalline chromosomes (which occurred after the loss of bulk nucleosomes and the acquisition of DVNP – see Janouskovec et al. 2018. PNAS. 114:E171-180) suggesting they play a role in chromosome structure.
Line 667-674: This information may be worth including in the introduction.
Line 682-684: Does this Hi-C model fit this data? Is this model reflected in the domains or pseudo-layers?
Line 684-690: It is interesting that chromosome morphology can be inferred from the Hi-C data but given that we already know dinoflagellate chromosomes form rods from microscopy data (as mentioned Line 672-673) is this more important as a validation of the Hi-C data?
Line 707: “layer” should be “layers”

Line 711-716: Is there any relation between the loops that have been described from microscopic studies and the putative loops described in the results (line 512-517, Figure 5G)?
Line 748: ‘bp_resolution” should be “bp-resolution”
Line 753: “is” should be “are”
Line 757-758: “symbiont” should be “symbionts”
Line 839/848: “dry ice more” should be “dry ice for more”
Line 875: “pellet” should be “pellets”
Line 1040: I think “Mb” should be “bp”?
In general I prefer “plastid” over “chloroplast” in these organisms, since the latter specifically means “green” plastids, and these are red-derived ones.

Reviewer #2:
Remarks to the Author:
The manuscript describes a characterization of the genome of the dinoflagellate S. microadriaticum and the organization of its genes. Authors then use Hi-C to determine the 3D organization of S. microadriaticum chromosomes and conclude that chromosomes are organized in domains separated by boundaries located at sites of convergent transcription.

The paper is well written and the data analyzed properly. Overall, this is a solid manuscript that clarifies questions in the field of dinoflagellate nuclear biology. I leave it up to the editor to decide whether the significance of the results raises to the expectations of Nat Genet.

Major comments

1. Page 8, lines 190-191 “Interactions between loci on either side of a boundary are strongly repressed, and therefore domain boundaries act as structural “insulators”. Please reconsider making this conclusion. There is no evidence in the data supporting this conclusion. It is equally possible that proteins bound to sequences within each domain prefer to interact with other proteins present in the same domain. It is possible that proteins in one domain do not “want” to interact with proteins in the adjacent domain, rather than they “are not allowed” to do so. The same concern applies to lines 199-200 “loci are identified that 200 strongly prevent interaction to occur across them”. The analysis only identifies a change in the directionality of interactions, but it is agnostic as to the cause.

2. Figure 2 and accompanying discussion. The authors go to great lengths to argue that, based on the decay of contact frequency with distance, the chromosomes have a rod-like structure. It is surprising that authors do not use evidence from microscopy to argue this point. Somebody must have looked at dinoflagellates under a microscope. Do the chromosomes look rod-like in structure?

3. Figure 2. Authors base their conclusions on the organization of S. microadriaticum chromosomes on the shape of P(s) by extrapolating information from decay curves in mammalian interphase and metaphase chromosomes. Authors should comment on whether this comparison is really appropriate. Mammalian chromosomes contain nucleosomes plus all the numerous multivalent complexes bound to different histone modifications, all of which are apparently missing in S. microadriaticum. In addition, mammalian nuclei contain cohesin and condensin, which contribute to chromosome architecture by forming loops and through the process of extrusion. These biophysical and biochemical processes in mammals must contribute to the particulars of distance decay curves, and their absence in S. microadriaticum may not allow one to extrapolate information from mammals to dinoflagellates.

4. Figure 3C and lines 317-319 “This provides further evidence that these chromatin domain boundaries and detected by Hi-C and their distinct sequence composition are bona fide chromosomal features”. This language may be too strong. The decrease in GC content goes from 0.50 to 0.45, which is 10%. Authors should mention the actual number in the text to avoid misleading the reader. Later on, lines 358-359, authors indicate that “gene density and expression were positively correlated with GC content”. Could expression and gene density, rather than GC content per se, be responsible for the formation of domain borders?

5. Figure 5D. It appears that transcription continues through the boundary. Is this because the location of the boundary is mapped with less than 5 kb resolution? Please comment in the text.

6. Unless the authors strongly disagree, I would suggest a change in the order of the topics discussed in the manuscript, talking about gene organization, TEs, GO functions, etc. first and ending with chromosome organization, with section “S. microadriaticum chromosomes are folded as linearly organized layered rods” last. Otherwise the most interesting part of the manuscript is at the beginning and the most boring at the end.

7. The significance of the conclusions would be higher if the results could be interpreted to gain understanding of general principles of chromosome organization. I imagine that S. microadriaticum has cohesin and/or condensin and that one or both are able to extrude. Could either complex accompany RNA polymerase and accumulate at the 3’ of convergent gene arrays, as it does in yeast, where it forms gene loops?

Minor comments

1. Introduction. “More recent transcriptome studies, however, confirmed that dinoflagellates do possess histones, but lack histone H1”. Dinoflagellates do have histone genes and they are transcribed but the actual histone proteins have only been identified very recently using mass spec, suggesting they

are translated at very low levels.

2. Introduction. “The liquid-crystalline conformation of dinoflagellates may represent a third chromosome folding state”. This idea appears to come for a 1972 paper in which the author studied sections of insect cuticle. There is one panel in one figure in which the author of the manuscript examines dinoflagellate nuclei. They do look funky, but I wouldn’t say they look like crystals. Same applies to the Discussion, lines 712-713, “banding pattern along liquid-crystalline chromosomes and decondensed loops emanating from the condensed core”. A superficial reading of the literature seems to now suggest that these were artefacts caused by fixation methods.

3. Page 8, lines 198-199 “to determine the positions of Hi-C domain boundaries
199 genome-wide at 10 kb resolution (Figure 1C)”. Figure 1C indicates the bin size is 50 kb.

4. It would help if authors include a high resolution panel of just one domain to be able to appreciate the substructure within the domain.

5. Page 14, lines 348-350, “gene density in S. microadriaticum ranges from 38 – 155 genes per Mb, showing a greater gene density compared to other eukaryotic genomes such as human (3.5 – 23 genes per Mb”. It may be more instructive to compare gene density with organisms such as yeast, C. elegans, Arabidopsis of Drosophila.

6. Page 15, lines 409-411, “S. microadriaticum has a relatively low number of repetitive elements, comprising only 26.5% of the genome compared to 37.5% in mouse, ~50% in human, and 84.7% in wheat”. As above, perhaps a comparison with yeast, C. elegans and Drosophila would be more appropriate. Do the LINEs encode a reverse transcriptase and do they have a similar organization to LINEs in humans or are they called LINEs because they are long and they don’t have LTRs?

7. Page 21, lines 520-521, “a strong reduction is observed at convergent boundaries”. Whether the observed reduction is strong or minor is subjective. Please replace “strong” and “minor” for the actual numbers and let the reader decide.

8. Discussion, lines 702-705. “We performed Hi-C using a combination of formaldehyde and DSG. The Hi-C interaction maps and P(s) plots obtained this way are very similar (Supplemental Figure S8), suggesting the small exponent is not due to low cross-linking efficiency”. Move to results?

Reviewer #3:
Remarks to the Author:

By combining Hi-C and PacBio sequencing, Nand, Zhan and colleagues assemble and provide the first version reference genome for the dinoflagellate Symbiodinium microadriaticum. Nowadays, de novo genome assembling is not technically challenging and even not particular interesting to some biologists. However, providing a reference genome for a species not previously sequenced is of great value to the scientific community, not to mention in this case, a very unique and important organism.

The authors then analyze the genomic sequence including GC content, gene density, gene orientation, repetitive elements and so on; routine and basic analysis. Many unique features are revealed. Gene density increases towards the ends of chromosomes. Genes are arranged in unidirectional blocks alternating between top and bottom strands. Genes involved in specific biological processes are enriched in some chromosomes. These genetic features are quite different from eukaryotic model organisms that are mostly used in laboratories.

Besides, the genome of S. microadriaticum is found being organized into structural domains, similar to topologically associating domains (TADs), and these domains are separated at sites where transcription of two gene blocks converge, suggesting a strong correlation between the linear genetic sequence and three-dimensional organization.

Dinoflagellates chromosomes exist in a unique state and are not packaged by histones, which is dramatically different from metazoans analyzed so far. Chromosomes in a special state provide a unique window for better understanding elusive genome folding mechanisms and function-structure relationship, for example, plant vs animal, inactive X chromosome vs autosomes, cells in M phase vs interphase, sperm vs somatic cells, and many other comparisons.

To me, this is a quite impressive work on a species that is not usually used for genetic study. Nevertheless, there are a few concerns that should be further clarified by the authors.

Major comments:

1. The reference genome assembling is a very important part of this work. However, a total of 183,768,579 bp were set aside, more than one fifth of the whole genome. These excluded sequences interact frequently with more than one chromosome (line 164-166). After clustering these sequences into 94 groups, the authors wrote “each of these clusters interacts particularly frequently with only one of the 94 chromosomes, and each chromosome interacts frequently with sub-scaffolds from only one such cluster” (Line 168-170). It seems to me that a specific sub-scaffold, if clustered in one specific cluster, then it is supposed to interact frequently with a specific chromosome. Since these clustered sub-scaffolds interact frequently with only one of the 94 chromosomes (Figure 1C), the authors may want to think about trying other technologies to see if these large amounts of sequences can be placed at proper locations in the genome assembly. This will surely improve the completeness of this reference genome,

and this should be doable.

2. The authors find that the genome of S. microadriaticum is partitioned into domain structures. In mammalian cells, chromosome compartments and TADs are lost in metaphase, and chromosome structure described for nonsynchronous cells is restricted to interphase (Naumova et. al., Science, 2013). In this paper, Hi-C maps of G2/M immobile and G1/S flagellated cells are compared, and no obvious differences revealed (Supplemental Figure S2, line 193-195). The authors should explain if cells of this specific species in G2 phase have the same very condensed chromosomes as in M phase and different from mammalian G2 phase, or vice versa. Or if through the whole cell cycle, the chromosomes are consistently in a non-changing state. Whichever it is, the authors may want to provide microscopic images to show if and how the genome/chromosomes changes through the cell cycle fluctuating between condensed and relaxed states as in mammalian cells. Otherwise, the comparison between G2/M and G1/S phase and the claim that the domain structure does not change seem not valid to me.

Minor comments:

1. The exclusion of sub-scaffolds does not affect the 3D genome architecture analysis (line 214-217), however, we cannot rule out the possibility that their distribution pattern in the genome may affect the GC content, gene density analysis, and also other analyses.

2. Compartmentalization of chromosomes is another structural feature of many eukaryotic genomes. It seems to me that compartment-like patterns can also been identified in the Hi-C heatmaps (Figure 1C). The authors may want to further analyze if domain structures preferentially interact within compartments as well in this species.

3. It is quite interesting that the domain borders are frequently found at the convergent sites of two unidirectional gene blocks. This reminds me of a work published showing that genes flanking borders were also found convergent in the Drosophila genome (Hou et. al., Mol Cell, 2012). Genes in the genome of Drosophia are not so neatly organized into unidirectional blocks as in S. microadriaticum. If this is just a coincidence or it implies a conserved mechanism can be discussed.

4. It would be better if mechanistic studies can be carried out to test if transcription is playing a role in the domain structure establishment. Given the extensive results reported in this article, this may not be a prerequisite for its publication.

Overall, this work is quite interesting to me. The results provide not only a valuable reference genome but also many discoveries of unique genetic and structural features in the genome of a special species. It may very likely open a window for people to better understand how the genome is organized from an

evolutionary, and/or function-structure related point of view.

Author Rebuttal to Initial comments

Response to reviewers

To all three reviewers:
We thank you for your very important comments. We have attempted to address all of them and believe this has greatly improved our manuscript. We address your comments below point-by-point. Here we first summarize briefly what the major changes are in the revised manuscript:

1) In response to reviewer 1 we have summarized how insights derived from our new Hi-C data compare to previous models for chromosome organization in dinoflagellates
We edited how we present our data in comparison to previous studies (new additions to the discussion). However, because our manuscript was quite significantly over the word limit we had to keep this as concise as possible. We anticipate future articles, e.g. reviews, will provide a much deeper integration of the various models and studies on dinoflagellate chromosomes performed over the last decades.

2) In response to reviewer 2 we have rearranged the order of the manuscript.
Following the suggestion of reviewer 2, in the revised manuscript we first present the assembly of the *S. microadriaticum genome*, then describe the genetic organization of the chromosomes (GC content, gene and repeat distributions), and then focus on the spatial organization of chromosomes (rod-shaped folding, chromatin domains related to unidirectional gene blocks, and effect of triptolide and DRB on domain folding).

3) In response to reviewer 3 we have further improved the assembly.
We generated new PacBio long-read based contigs and used these for extensive gap filling to produce a new assembly: Smic1.1N. In this new assembly we have added >110 Mb of DNA, and were able to place on assembled chromosomes more than 50% of the ~180 Mb we could not place before. We believe this addresses a major concern raised by this reviewer. We note that by incorporating new sequences that are often highly repetitive (as we had shown in the first version of the manuscript), we also introduced new complications (illustrated in the updated Extended Data Fig. 2). In the revised manuscript we describe the original assembly Smic1.0 that is extensively manually curated, and is a very conservative but reliable scaffold with gaps,

as well as the newly gap-filled assembly Smic1.1N that contains more sequences but is not manually curated. We performed all analyses on both assemblies with almost identical results.

4) In response to reviewer 3 we have analyzed the effect of blocking transcription on domain formation

We have treated cells with two chemicals known to block transcription in other eukaryotes. This treatment led to growth arrest. However, Hi-C data show that this treatment also led to significant changes in chromosome folding, with domain boundaries disappearing. These new exciting data are shown in the newly added Figure 6. We were not able to identify conditions where S*. microadriaticum* takes up modified bases such as EU, and therefore we were not able to ascertain that this treatment indeed blocks nascent transcription. Based on the data shown here we can conclude that chromosome conformation can be modulated under conditions that block cell growth, *possibly* through effects on transcription. Importantly, the fact that domain formation is sensitive to growth conditions provides further evidence that domain boundaries are not due to remaining genome assembly errors.

Below we present a point-to-point response to all comments made by the reviewers

Reviewer #1:

Remarks to the Author:
In this manuscript, Nand et al. use a combination of DNA sequencing methodologies to generate a chromosome-scale genome assembly for the dinoflagellate Symbiodinium microadriaticum, which they use to investigate three-dimensional genome architecture and its relation to genomic features. This represents a major feat that not only provides an invaluable resource for studying dinoflagellate genomics, but new insights into the biology of dinoflagellate chromosomes. Despite its importance, progress in understanding dinoflagellate chromatin biology has slowed, and this paper exemplifies how interdisciplinary approaches can move the field forwards.
In general, I found the paper to be well written, the results robust and thought-provoking. I have to note that I am not an expert in Hi-C, but the methods appear robust and echo the highly regarded work of the labs involved.
In general, my criticisms centre on making the manuscript more interpretable for a wider audience (particularly those not familiar with Hi-C and dinoflagellates) and integrating additional dinoflagellate concepts and literature. I also had some concerns about the gene ontology analysis. These should all be addressable through a few minor revisions to the text and analyses.

I feel the manuscript could benefit by better integrating concepts in dinoflagellate chromatin biology and genomics with the Hi-C methodology. The introduction needs more information on dinoflagellate chromosome structure and Hi-C (which chromatin biologists and protistologists may be less familiar with, respectively). We know a reasonable amount about the morphology of dinoflagellate chromosomes from microscopy studies (e.g., their linear/rod shaped conformation, the presence of banding and ridges, and the existence of loops which may be transcriptionally active) and replacement of bulk nucleosomes by DVNP and (in core dinoflagellates) HLPs, should be noted here. This would help place the results in context.
It would also be interesting during the discussion to talk more about current hypotheses of dinoflagellate chromosome structure (as was briefly done – lines 674-679) and discuss how they align with the Hi-C data. Which parts of these hypotheses agree with the Hi-C data? Which are refuted? What kind of Hi-C data would you expect from dinoflagellate chromosomes? What are the limits of the Hi-C data? Overall, I think this manuscript would be significantly more accessible if it included a detailed summary diagram depicting what this study has revealed about dinoflagellate chromosome structure and function.

Response: We thank the reviewer for the supportive comments. We have extensively revised the manuscript and in both the introduction and the discussion we clarified better how our new insights relate to earlier studies. Specifically, in the discussion we added a paragraph that summarizes what our study shows:
1. divergent gene blocks form structural domains
2. Terminal domains are distinct with higher local interactions
3. Chromosomes are relatively stiff rods, as has been seen in earlier imaging-based studies
4. No classical compartmentalization is observed, in contrast to other eukaryotes
5. no circularity or helical coiling is observed, in contrast to earlier models
6. No specific point-to-point loops are detected, in contast to other eukaryotes
9. Transcription appears important for domain formation (with experimental caveats), as in prokaryotes

We also note that the relation of the domains seen in Hi-C to the arches seen in electron micrographs is not clear and we prefer not to speculate about this interesting topic without additonal imaging data. We hope that we can study this topic in more detail in the near future.

Other comments:
Line 1: Is S. microadriaticum a coral endosymbiont? I only know it from jellyfish (e.g., Cassiopeia).

Response: Yes, it is. Apart from this specific strain being reported to have been isolated from the coral *Stylophora pistillata* from Aqaba, there are several studies based on large scale

sampling and high throughput ITS2 screens across the Red Sea showing that *S. microadriaticum* is found as the main symbiont of several coral species (Ziegler et al., 2017; Voolstra et al. 2020; Osman et al., 2020; Hume et al.; 2020). Thus, although originally characterized as a symbiont from jellyfish, it is abundantly associated with corals from the Arabian Seas.

Cited References:

Ziegler M, Arif C, Burt JA, Dobretsov S, Roder C, LaJeunesse TC, Voolstra CR (2017) Biogeography and molecular diversity of coral symbionts in the genus Symbiodinium around the Arabian Peninsula. J Biogeogr 44:674–686

Hume BCC, Mejia-Restrepo A, Voolstra CR, Berumen ML (2020) Fine-scale delineation of Symbiodiniaceae genotypes on a previously bleached central Red Sea reef system demonstrates a prevalence of coral host-specific associations. Coral Reefs 39:583–601

Osman EO, Suggett DJ, Voolstra CR, Pettay DT, Clark DR, Pogoreutz C, Sampayo EM, Warner ME, Smith DJ (2020) Coral microbiome composition along the northern Red Sea suggests high plasticity of bacterial and specificity of endosymbiotic dinoflagellate communities. Microbiome 8:8

Voolstra CR, Buitrago-López C, Perna G, Cárdenas A, Hume BCC, Rädecker N, Barshis DJ (2020) Standardized short-term acute heat stress assays resolve historical differences in coral thermotolerance across microhabitat reef sites. Glob Chang Biol 26:4328– 4343

Line 26: See Line 1.Line 50: “enables” should be “enable”

Response: Changed accordingly.

Lines 71-76: This makes it sounds like dinoflagellates are neither eukaryotes nor prokaryotes (hearkens back to the incorrect idea that dinoflaglellates are ‘mesoeukaryotes’). This should be revised to avoid confusion.

Response: We understand the concern of the reviewer and have changed the sentence accordingly to avoid confusion. The revised sentence reads: “The liquid-crystalline conformation of dinoflagellates may represent a third chromosome folding state, in addition to the typical

nucleosomal form commonly found in eukaryotes and the supercoiled circular form in most bacteria."

Line 86: "or" should be "and"

Response: Changed accordingly.

Line 130-135: Can you add average or median chromosome sizes (or sizes of the assembled chromosome clusters)?

Response: We thank the reviewer for this suggestion. In the revised manuscript we mention the size range of chromosomes, and the median size for the manually curated Smic1.0 assembly and the gap-filled Smic1.1N assembly.

Line 174-175: This could use slightly more clarification. Why does copy number result in more inter-chromosomal interactions? Is it because at a small frequency each genomic region interacts with other chromosomes and when you collapse a multicopy region into a single bin, the small number of interactions sums to be noticeable?

Response: We thank the reviewer for pointing out that we did not explain this carefully. There are various reasons why increased copy number can alter inter-chromosomal interactions. First, if the additional copies are all located at the same genomic location (e.g. as a tandem repeat) and in the assembly these are collapsed into a single copy/bin, then we often see that inter-chromosomal interactions as detected by Hi-C appear too high. This is exactly for the reason the reviewer suggests. However, in many of the cases we observe here we believe the scenario is different: the additional copies of a locus are located at different places in the genome. If the assembled genome only contains this sequence on _one_ chromosome (i.e. the other copies on other chromosomes are not included in the assembly), then in Hi-C strong inter-chromosomal interactions will be observed between the genomic locus containing the included copy, and the other chromosomes that contain the same sequence but that were not included in the assembly. We now clarified this issue in the revised manuscript. We added the following statement:

"Increased Hi-C interactions between loci located on different chromosomes can occur when sequences are present in multiple copies at a single location or are present at multiple locations, but are included only once at one location in the assembly. Analysis of Hi-C read coverage indicated that while sequences present on the assembled chromosomes 1-94 are all present at

similar copy number, many of the sub-scaffolds that were set aside were present at much higher copy number (on average 11 times higher than sequences included on the chromosome scaffolds; Fig. 1B)."

Line 177: Where in the chromosomes are these regions? Is it consistent between chromosomes? Given that each chromosome has one of these high copy regions, could they have functional significance (e.g., could they act as centromeres?)?

Response: The cluster sequences are enriched in repetitive sequences, which likely contributed to our inability to place many of the unambiguously to specific genomic locations. We note that each cluster is composed of a collection of sequences that all interact most frequently with 1 specific chromosome. However, it is not the case that each chromosome has only 1 high copy region: the sequences within a single cluster appear to be located at multiple locations along each chromosome. Therefore, we believe these are interspersed repetitive sequences that can be located all along the chromosomes, and it is unlikely they represent a single centromere. We further point out that we have now been able to fill many more gaps in the original Smic1.0 assembly and in the process we were able to place more than half of the cluster sequences on chromosomes.

Line 186-195: This paragraph should be revised as it is unclear whether this is an introduction to interpreting Hi-C heatmaps generally or an interpretation of the data itself. At first I thought it was about the data, but then I saw the citation and no figure references and thought it was an introduction, and then it concluded somewhat abruptly with the comment on coccoid vs mastigote cells.

Response: We apologize for not being clear. This paragraph indeed detailed the Hi-C approach and data described in this paper, shown in Figure 1. The paragraph describes the new finding that each chromosome is composed of a series of domains, and that this is observed in both mastigote and coccoid-enriched cultures. The reference in the text referred to a publication where we first introduced the concept of insulating domain boundaries in *C. elegans*. In the revised manuscript we have rewritten this important paragraph, and explicitly refer to Figure 1 to make clear it describes new results reported in the current work.

We replaced this paragraph with this statement:
"The Hi-C interaction maps of all chromosomes show domainal features (Fig. 1C): each chromosome displays a series of square-shaped domains along the diagonal with relatively elevated interaction frequencies within them and lower frequencies between them. The

boundaries between them are often, but not always, sharp transitions. Interaction frequencies between loci on either side of a boundary are strongly reduced as compared to interactions between loci not separated by a boundary. Further, interactions between these Hi-C domains form a series of squares and rectangles farther from the diagonal. Hi-C interactions maps obtained from cultures enriched in coccoid cells (G2/M immobile cells) or mastigote cells (G1/S flagellated cells) revealed no obvious differences (Extended Data Fig. 3).

Line 202-203: Does “on most chromosomes” refer to “on canonical chromosomes”?

Response: “on most chromosomes” refers to the fact that the computational analysis missed a set of weak domain boundaries, and visual inspection indicated that “most of the chromosomes” had some boundaries that could be discerned by eye, but that were not identified using the conservative algorithm we employed.

Figure 1B: The interactions between clusters 1-94 and other chromosomes, besides their main interacting chromosome, are very hard to see which makes it a bit confusing when reading the results. This could be a resolution issue.

Response: The reviewer is correct, this is mostly a resolution and color scale issue. Both resolution and color scale were chosen to fit the large whole-genome chromatin interaction map. When zoomed in, or if the color scale is adjusted, one can see the high level of interactions with multiple chromosomes.

Figure 1C: I think the edges of the plots should be labeled (e.g., chromosome vs chromosome and chromosome vs cluster)

Response: In the revised manuscript we now include a new and more inclusive genome assembly (Smic1.1N) that incorporates a very significant fraction of the DNA (~56%) that on the clusters in Smic1.0. Now that many of the cluster sequences are incorporated in the genome assembly Smic1.1N, we have reduced the focus on clusters in Figure 1, removed the cluster interaction maps in Figure 1C, and updated Extended Data Fig. 2 (was Supplemental Figure 3 in the previous submission) that now shows Smic1.1N interaction maps.

Line 219-241: I understand the interpretation that the third regime in the P(s) implies a linear chromosome but I think it’s worth elaborating on the concept of pseudolayers. Do pseudolayers

refer to the fact that within the rod, the genome is not significantly mixed along the vertical axis of the chromosome? If each locus interacts with regions 3Mbp upstream and downstream, it is unclear to me what the size of a pseudolayer refers to as it seems like the interactions should be a continuum across the chromosome. Could the size of regime II reflect other features of the chromosome such as its width?

Response: The reviewer brings up a very important point. Pseudolayers are referred to as “pseudo” exactly because they do not have defined boundaries. Each locus has only a limited ability to mix and interact with other loci along the length of the chromosome: it can interact with loci located up to 3 Mb up- and downstream of it, but not beyond. We have referred to this range as a pseudolayer in our earlier work on rod-shaped mitotic chromosomes (Naumova et al. Science 2013 and Gibcus et al. Science 2018). We note that if loci can mix freely all along the entire long axis of a rod-shaped chromosome the P(s) plot would not show regime III. In a completely equilibrated scenario this would lead to a plateau in P(s), as we described in Lieberman-Aiden et al. Science 2009. Importantly, the term “pseudo-layer” is not widely used, and also not required for explaining our results here. Therefore, in the revised manuscript we no longer use this term.

The reviewer also brings up how to interpret regime II. In the past we have shown that for mitotic chromosomes this regime reflects how DNA is folded within a cross-section of rod-shaped chromosomes. We have learned from such analysis that this regime can tell us about the sizes of chromatin loops in mitotic chromosomes of human, mouse and chicken cells. The sizes of the loops are indeed predictive of the diameter of the mitotic chromosome, as the reviewer suggests. We could interpret this for metazoa because we know, from other studies, that mitotic chromosomes in those organisms fold as arrays of loops and thus we could build polymer models for such configurations to test which combination of parameters (e.g. loop size) would reproduce the experimentally observed P(s). However, how to interpret regime II for dinoflagellates is not known because we do not know whether dinoflagellate chromosomes form similar loop arrays. Interpretation of P(s) for regime II will therefore need to await further studies of the internal folding of chromatin within chromosomes, most likely using microscopic observations.

Line310: Can you show the trend in GC for all chromosomes or averaged across chromosomes?

Response: in Figure 2 of the revised manuscript (was Figure 3 in the first submission) we show the GC-content trends as a function of genomic distance to the telomere averaged over all chromosomes. We hope this addresses the reviewer’s comment.

Line 318: Remove ‘and’

Response: Changed accordingly.

Line 341: “allows to elucidate” should be “allowed us to elucidate”

Response: Changed accordingly.

Line 343: “particular” should be “particularly”

Response: Changed accordingly.

Line 343: “have” should be “have been”

Response: Changed accordingly.

Line 356-357: Consider rewording as “resulting in a moderate yet significant positive correlation between gene density and gene expression (0.33 R2, 4.2e-157 p-value)

Response: Changed accordingly.

Line 359: Figure 4H is cited before other panels in Figure 4. It should be revised to keep things in order.

Response: Changed accordingly. We have edited to text so that discussion of the correlations shown in panel H is now at the end of the corresponding section of the results.

Line 359-360: Could the correlation between gene density and GC reflect GC codon bias in dinoflagellates (e.g., see Williams et al. 2017. Mar Drugs. 15(5): 125). Also isn’t it intuitive that more RNA-seq reads should map to a region if there are more genes in it? It would make sense

to also look at expression level across the chromosome (i.e., where are the highest expressed genes and what genomic features do they correlate with?).

Response: The reviewer is correct that a higher gene density will result in more RNA-seq reads mapping to the corresponding genomic region. However, our correlation analyses in Figure 3H only shows a weak positive correlation of RNA-seq reads and gene density.

Figure 4A: Add genomic reads (e.g., like in Figure 1B) to these plots to show that increases in transcription are not a reflection of repeats and high copy number.

Response: this is an excellent suggestion. We investigated this, and as shown in Figure 1B, the read coverage along the assembled chromosomes is very even and no enrichment is observed near the telomeres.

Figure 4H: The figure is missing negative values on the legend.

Response: We fixed this in the revised manuscript.

Line 399: Except for some eukaryotes, like kinetoplastids, where there are large co-oriented gene arrays.

Response: We agree with the reviewer and changed the sentence accordingly. The revised sentence reads:

"Such pattern where immediate neighboring genes are more likely to follow the same orientation is commonly observed in prokaryotes, while in most eukaryotes orientation of neighboring genes are less or not correlated. Notable exceptions include kinetoplastids that also show blocks of unidirectional genes".

We also mention Trypanosomes briefly in the revised discussion.

Line 414: Why is it surprising?

Response: SINEs and LTRs are usually very common in eukaryotes and can make up a sizable portion of the genome, such as in mammals where they account for >10% of the genome. The respective sentence has been changed accordingly, it now reads:

“Surprisingly, the genome is practically devoid of short interspersed nuclear elements (SINEs) and long terminal repeats (LTRs), together accounting for less than 1% of the genome, which is in stark contrast to many other eukaryotes where these elements can make up significant amounts of the genome”.

Line 467-469: It is unclear what Figure 5B is showing and what the analysis indicates. Please elaborate and add labels to the plot.

Response: As indicated in the legend, Figure 5B shows a pile-up (average) of the Hi-C interactions around the 441 identified boundaries to provide a higher resolution view of the interactions around these boundaries. The plot clearly shows that interactions across boundaries are strongly depleted (upper right and lower left quadrants) i.e. that the DNA on the opposite sites of the boundaries rarely interact, thereby forming the boundaries visible in the Hi-C interaction maps. We apologize for not having added labels to the legend and we have corrected this now.

Line 488: “converges” should be “converge”

Response: Changed accordingly.

Line 524-573: Given the nature of the tandem repeats I find the gene ontology analysis looking for functional enrichment in chromosomes somewhat problematic. My main concern is that a large tandem array of a single gene could make it appear that a chromosome is enriched for a certain process. For example, chromosome four is apparently enriched for photosynthesis related genes but to what extent is this just a result of having six duplicated copies of ATP synthase subunit C? In other words, a chromosome may have lots of photosynthesis genes but only a marginal proportion of the photosynthesis pathway. Was this accounted for in the analysis (it was unclear from the methods)? If not, it would be worth trying to normalize for this. Perhaps this could be done by assigning genes to KEGG terms and looking for enrichment of pathway components (e.g., if there are X photosynthesis KOGs in the genome, can you reject the hypothesis that they are distributed randomly?). Another approach could be to collapse

recent paralogs and duplications into single data points based on BLAST E-values or percent identity

Response: Thank you for bringing this up. This is a point that we had discussed among the authors. To directly answer your question: no, we decided against 'normalizing' the enriched genes. Rather, we argue that any (substantial) enrichment of process-related genes is still an enrichment for that process. Arguably, enrichment of a diversity of genes or just a distinct gene from a given pathway had to be positively selected at one point (and retained in subsequent evolutionary time), which argues for its functional significance. For instance, studies have argued in the past that functionally important genes are oriented in tandem-arrays in dinoflagellates (Stephens et al. BMC Biology 2020; Aranda et al. Scientific Reports 2016, Lin et al. Science 2015) and we think what we are seeing is (partially) consistent with this. So, yes: in the majority of cases we see distinct enrichment of a particular gene (which *sensu stricto* would argue that not the entire process, but this gene is enriched), but we argue that this may be biologically and functionally as significant as enrichment of pathways of genes. We actually specifically tested for enrichment of entire pathways, which we could not confirm (we were very excited about this initially, as it reminded us of the operon structure in bacteria). We have added text to the Discussion section to reflect that enrichment is largely seen for specific genes, but by the nature of the analysis, it's depicted in the form of pathways.

Added text reads: "Notably, in the majority of cases we found duplications of specific genes (rather than for sets of genes encoding for entire pathways), which in the course of the Gene Ontology enrichment analysis are portrayed as pathway enrichments. While this is a limitation of our analysis, it reflects the notion that specific genes of specific pathways are enriched (rather than all genes of a given pathway), although the biological significance as to why some genes (of a given pathway) are enriched, while others are not, remains elusive at this point."

Line 524-525: More analysis of the tandem gene arrays would be worthwhile. For example, do the genes within an array tend to be related to a certain function? What about within chromosomes domains? Are genes within an array more closely related (e.g., recent paralogues) or are they more like operons, containing sets of functionally related genes? Based on line 527-529, it seems some of these questions may have been looked at but it was unclear where the data and results were.

Response: As we mention in the text we did a general functional enrichment analysis of the cluster sequences and found them to be enriched in features associated with mobile elements. In the revised manuscript we describe a more inclusive assembly (Smic1.1N) that incorporates a significant fraction of these cluster sequences in the 94 chromosomes. Therefore, in the

revised manuscript we no longer analyze the cluster sequences separately beyond the general analysis mentioned above.

Line 604-605: “of the unique clade of dinoflagellates,” should be “of the dinoflagellates.”

Response: Changed accordingly.

Line 610-615: Are the interactions between activating and inactivating domains in other eukaryotes dependent on the more homogenous nature/flexibility of interphase chromosomes? Since dinoflagellate chromosomes are permanently condensed, is it expected that there wouldn’t be these interactions? Interesting to think about what functional consequences or coping mechanisms there may be without these interactions.

Response: The reviewer brings up a very good point. In rod-shaped chromosomes the preferred long-range interactions between sets of active domains, and between sets of inactive domains would indeed be difficult to accommodate. In mitotic chromosomes of human, mouse and chicken we also do not see such preferred long-range interaction, possibly due to the stiffness of mitotic chromosomes. In the revised manuscript we add this very important possibility. However, we note that we do not see such preferred interactions between_ chromosomes either, while in interphase chromosomes of mammals and birds this is readily detected. We further note that in mammals these preferred interactions are thought to be driven by histone modifications (Gibson et al. Cell 2019), and dinoflagellate chromatin is mostly devoid of histones. In the revised manuscript we have clarified this issue in the discussion.

Line 640-642: What about in Trypanosomes where there are large arrays of co-oriented genes? Has any correlation with domain structure been observed there? (See Muller et al. 2018. Nature 563:121-125)

Response: We thank the reviewer for pointing out that there are some similarities to the organization of genes in Trypanosomes. It has been shown before that in Trypanosomes genes are organized in unidirectional blocks and that they are transcribed from a single TSS. In some cases these are in a divergent orientation, but such blocks can also be in tandem. This is similar to what we now describe for *Symbiodinium*, where genes are also organized in alternating unidirectional blocks. In Muller et al. Nature 2018 it is shown that terminal domains form more compact chromatin structures, and again that is also what we find for dinoflagellates. However,

there are very important differences between dinoflagellate chromosome organization and what has been described for Trypanosomes: First, in Trypanosomes there is no relation between unidirectional gene blocks and chromatin domains we observe for dinoflagellates. Second, while terminal domains in both Trypanosomes and dinoflagellates show elevated chromatin interactions, the mechanism and functional relevance for this phenomenon are likely very different because in Trypanosomes these domains are compact and transcriptionally silent but in dinoflagellates these are the most highly expressed regions of chromosomes. Third, in Trypanosomes the most active genes are in the middle portions of the chromosomes (without Hi-C domain formation at convergent sites), while in dinoflagellates, gene expression (with Hi-C domain formation at convergent sites) occurs across the length of the chromosomes (with expression the highest in terminal regions). In the revised manuscript we now describe the interesting similarities and clear differences between chromosome organization in Trypanosomes and dinoflagellates.

Line 644-646: This is speculation, but given the similarities between genome conformation in Caulobacter and S. microadriaticum, it is interesting to wonder whether domains are in part defined by HLPs which dinoflagellates derived from bacteria through horizontal gene transfer. The acquisition of HLPs actually correlates with the origin of liquid crystalline chromosomes (which occurred after the loss of bulk nucleosomes and the acquisition of DVNP – see Janouskovec et al. 2018. PNAS. 114:E171-180) suggesting they play a role in chromosome structure.

Response: the reviewer raises several really exciting possibilities related to the role of HLPs and DVNPs in organizing chromatin in dinoflagellates. We are very interested in exploring this in the future, but our current data do not really allow us to speculate on their functions in the current manuscript.

Line 667-674: This information may be worth including in the introduction.

Response: In the revised manuscript we have edited the introduction and discussion extensively to introduce relevant previous models in the introduction and place our data in the context of these models in the discussion. We hope this addresses the reviewer's comment.

Line 682-684: Does this Hi-C model fit this data? Is this model reflected in the domains or pseudo-layers?

Response: It is currently not clear how our description of a layered organized related to models of cholesteric liquid crystal formation. Future imaging studies may shed light on this matter.

Line 684-690: It is interesting that chromosome morphology can be inferred from the Hi-C data but given that we already know dinoflagellate chromosomes form rods from microscopy data (as mentioned Line 672-673) is this more important as a validation of the Hi-C data?

Response: The reviewer is correct that the Hi-C data, and our interpretation that chromosomes are rods, is entirely consistent with prior microscopy data. This can be seen as validation of the Hi-C data. In the revised manuscript we make more clear that the fact that dinoflagellate chromosomes are rods was already known, and that our Hi-C data confirms this.

Line 707: “layer” should be “layers”

Response: Changed accordingly.

Line 711-716: Is there any relation between the loops that have been described from microscopic studies and the putative loops described in the results (line 512-517, Figure 5G)?

Response: We now briefly mention these previously described loops in the revised discussion.

Line 748: ‘bp_resolution” should be “bp-resolution”

Response: Changed accordingly.

Line 753: “is” should be “are”

Response: Changed accordingly.

Line 757-758: “symbiont” should be “symbionts”

Response: This sentence is no longer in the revised manuscript.

Line 839/848: “dry ice more” should be “dry ice for more”

Response: Changed accordingly.

Line 875: “pellet” should be “pellets”

Response: Changed accordingly.

Line 1040: I think “Mb” should be “bp”?

Response: Changed accordingly.

In general I prefer “plastid” over “chloroplast” in these organisms, since the latter specifically means “green” plastids, and these are red-derived ones.

Response: we changed “chloroplast” to “plastid”

Reviewer #2:
Remarks to the Author:
The manuscript describes a characterization of the genome of the dinoflagellate S. microadriaticum and the organization of its genes. Authors then use Hi-C to determine the 3D organization of S. microadriaticum chromosomes and conclude that chromosomes are organized in domains separated by boundaries located at sites of convergent transcription.

The paper is well written and the data analyzed properly. Overall, this is a solid manuscript that clarifies questions in the field of dinoflagellate nuclear biology. I leave it up to the editor to decide whether the significance of the results raises to the expectations of Nat Genet.

Major comments

1. Page 8, lines 190-191 “Interactions between loci on either side of a boundary are strongly repressed, and therefore domain boundaries act as structural “insulators”. Please reconsider making this conclusion. There is no evidence in the data supporting this conclusion. It is equally possible that proteins bound to sequences within each domain prefer to interact with other proteins present in the same domain. It is possible that proteins in one domain do not “want” to interact with proteins in the adjacent domain, rather than they “are not allowed” to do so. The same concern applies to lines 199-200 “loci are identified that 200 strongly prevent interaction to occur across them”. The analysis only identifies a change in the directionality of interactions, but it is agnostic as to the cause.

Response: We greatly appreciate that the reviewer brought up this important issue. The reviewer is entirely correct that we have no insights into the mechanism of boundary formation. It can be either active insulation (loci are not allowed to interact across the boundary) or loci within one domain somehow prefer to interact with each other rather than with loci in other domains. Our usage of the term “insulator” was based on our experience in mammalian cells, where some domain boundaries are indeed insulators (deleting them will lead to adjacent domains mixing with each other) and the mechanisms by which they insulate are known (CTCF protein blocking dynamic loop extrusion by the cohesin complex). However, as the reviewer rightly points out, we do not know what the mechanism of boundary formation is in dinoflagellates and, therefore, we have removed reference to boundaries as “insulators” throughout the manuscript.

2. Figure 2 and accompanying discussion. The authors go to great lengths to argue that, based on the decay of contact frequency with distance, the chromosomes have a rod-like structure. It is surprising that authors do not use evidence from microscopy to argue this point. Somebody

must have looked at dinoflagellates under a microscope. Do the chromosomes look rod-like in structure?

Response: The reviewer is entirely correct: previous microscopic analysis of dinoflagellate chromosome revealed elongated highly condensed crystalline structures. Our Hi-C data are consistent with this observation. In the revised discussion we refer to prior microscopic observation in more detail and highlight that our Hi-C data, and our interpretation of P(s) plots are consistent with earlier microscopic observations.

3. Figure 2. Authors base their conclusions on the organization of S. microadriaticum chromosomes on the shape of P(s) by extrapolating information from decay curves in mammalian interphase and metaphase chromosomes. Authors should comment on whether this comparison is really appropriate. Mammalian chromosomes contain nucleosomes plus all the numerous multivalent complexes bound to different histone modifications, all of which are apparently missing in S. microadriaticum. In addition, mammalian nuclei contain cohesin and condensin, which contribute to chromosome architecture by forming loops and through the process of extrusion. These biophysical and biochemical processes in mammals must contribute to the particulars of distance decay curves, and their absence in S. microadriaticum may not allow one to extrapolate information from mammals to dinoflagellates.

Response: The interpretation of P(s) does not really depend on the details of chromatin at the resolution of nucleosomes. First, this interpretation applies to any polymer (i.e. this has been done similarly, and successfully, for bacteria and eukaryotes in the past). Second, the scale at which we interpret P(s), hundreds of kB up to several Mb is at a scale that is several orders of magnitude larger than the level of nucleosomes. Therefore, we believe polymer theoretical interpretation of P(s) is appropriate here. We agree with the reviewer that at smaller scales, i.e. sub-kb up to several kb, the interpretation of Hi-C data for dinoflagellates must be done in a different way than for “normal” eukaryotes that do have nucleosomal DNA. However, we do not interpret Hi-C data at such fine scale here. Finally, we point out that *S. microadriaticum* does express condensins and cohesins, and these complexes most likely do form loops along dinoflagellate chromosomes, as we suggest based on data in Figure 5.

4. Figure 3C and lines 317-319 “This provides further evidence that these chromatin domain boundaries and detected by Hi-C and their distinct sequence composition are bona fide chromosomal features”. This language may be too strong. The decrease in GC content goes from 0.50 to 0.45, which is 10%. Authors should mention the actual number in the text to avoid misleading the reader. Later on, lines 358-359, authors indicate that “gene density and

expression were positively correlated with GC content". Could expression and gene density, rather than GC content per se, be responsible for the formation of domain borders?

Response: the reviewer is correct: the dip in GC content is only around 10%. In the revised manuscript we now mention this explicitly and we include the numbers in the text.

The reviewer raises a second point: can gene expression and gene density be responsible for the borders. In the revised manuscript, and in response to a specific request by reviewer 3, we have now included an experiment where we attempted to block transcription. This led to loss of domain borders as determined by Hi-C. Note that the chemicals we used for this experiment (DRP and triptolide) are well known for blocking transcription in human and mouse cells, but we could not determine whether transcription was indeed blocked in *S. microadriaticum* due to our inability to establish experimental protocols for nascent transcript detection in this species. Still, the treatment led to reversible growth arrest and loss of domain boundaries, indicating these structures are malleable and depend on cell state.

5. Figure 5D. It appears that transcription continues through the boundary. Is this because the location of the boundary is mapped with less than 5 kb resolution? Please comment in the text.

Response: the reviewer is correct, this is the result of the limited precision with which we could determine the position of the boundary (10 kb). We mention this is the revised manuscript (legend on Figure 5).

6. Unless the authors strongly disagree, I would suggest a change in the order of the topics discussed in the manuscript, talking about gene organization, TEs, GO functions, etc. first and ending with chromosome organization, with section "S. microadriaticum chromosomes are folded as linearly organized layered rods" last. Otherwise the most interesting part of the manuscript is at the beginning and the most boring at the end.

Response: This is an excellent suggestion. We have re-organized the entire results section according to the reviewer's suggestion.

7. The significance of the conclusions would be higher if the results could be interpreted to gain understanding of general principles of chromosome organization. I imagine that S. microadriaticum has cohesin and/or condensin and that one or both are able to extrude. Could

either complex accompany RNA polymerase and accumulate at the 3' of convergent gene arrays, as it does in yeast, where it forms gene loops?

Response: In the revised manuscript we have edited the introduction and discussion extensively to introduce relevant previous models in the introduction and place our data in the context of these models in the discussion. We also extended the discussion on possible roles for condensin and cohesin. However, in the absence of direct experiments testing roles for specific factors we feel we should be a bit restrained in speculating on their roles.

Minor comments

1. Introduction. "More recent transcriptome studies, however, confirmed that dinoflagellates do possess histones, but lack histone H1". Dinoflagellates do have histone genes and they are transcribed but the actual histone proteins have only been identified very recently using mass spec, suggesting they are translated at very low levels.

Response: The level of histones is very low indeed. However, the fact that dinoflagellates maintain histones (over a very long evolutionary time), and also express an arsenal of histone modifying enzymes indicates that histones do play some/a role. For our study the key point is that the bulk of DNA is not nucleosomal, but specific sites in the genome may contain one or a few nucleosomes, perhaps in a way similar to sperm chromatin in mammals.

2. Introduction. "The liquid-crystalline conformation of dinoflagellates may represent a third chromosome folding state". This idea appears to come for a 1972 paper in which the author studied sections of insect cuticle. There is one panel in one figure in which the author of the manuscript examines dinoflagellate nuclei. They do look funky, but I wouldn't say they look like crystals. Same applies to the Discussion, lines 712-713, "banding pattern along liquid-crystalline chromosomes and decondensed loops emanating from the condensed core". A superficial reading of the literature seems to now suggest that these were artefacts caused by fixation methods.

Response: The hypothesis that dinoflagellate chromosomes have properties resembling liquid crystals is based on several observations that chromosome exhibit birefringence (or birefractive) optical properties in polarized light. A good summary of the literature can be found in this recent review article: J.T.Y Wong (2019) "Architectural Organization of Dinoflagellate Liquid Crystalline Chromosomes. Microorganisms 22;7(2):27. Also, DNA is known to be able to form liquid crystals in vitro and when such crystals are examined by electron microscopy they look remarkably similar to electron micrograps of dinoflagellate chromosomes: Rill et al. (1989)

“Electron microscopy of liquid crystalline DNA: direct evidence for cholesteric-like organization of DNA in dinoflagellate chromosomes. Chromosoma 98(4):280-6. Therefore the idea that dinoflagellate chromatin has liquid crystalline features has been supported by several independent observations, but it of course remains a hypothesis.

The reviewer also suggests the banding patterns seen by others in the electron microscope can be due to fixation artefacts. This may well be the case, but this is not something we can address in our current manuscript.

3. Page 8, lines 198-199 “to determine the positions of Hi-C domain boundaries
199 genome-wide at 10 kb resolution (Figure 1C)”. Figure 1C indicates the bin size is 50 kb.

Response: This statement is correct: the Hi-C map shown is binned at 50 kb, but we calculated the insulation profile using Hi-C data binned at 10 kb. The Hi-C map is most illustrative at 50 kb resolution (given the frequent presence of repeated sequences that are not mappable by Hi-C). We decided to use Hi-C data binned at 10 kb resolution so that we could be more precise in defining the positions of domain boundaries.

4. It would help if authors include a high resolution panel of just one domain to be able to appreciate the substructure within the domain.

Response: this is a very good suggestion. We do not observe a clear pattern within the domains. In the revised manuscript we added zoom-ins at 5 kb resolution for each of the three chromosomes we show. In addition, in the new Figure 6 we now show a zoomed in section of chromosome 19 at 10 kb resolution. There appears little internal organization of domains. Finally, in GEO we make publicly available Hi-C matrixes at 1 kb resolution.

5. Page 14, lines 348-350, “gene density in S. microadriaticum ranges from 38 – 155 genes per Mb, showing a greater gene density compared to other eukaryotic genomes such as human (3.5 – 23 genes per Mb”. It may be more instructive to compare gene density with organisms such as yeast, C. elegans, Arabidopsis of Drosophila.

Response: from an evolutionary point of view dinoflagellates are as distantly related to yeast as to humans. Gene density in Dinoflagellates is similar to that in Drosophila and we added that comment to the revised manuscript.

Yeast: The gene density for the complete genome is 1 gene every 2,500 bp = 400 genes/Mb

C. elegans: 1 gene every 4,800 bp = 208 genes/Mb

Arabidopsis: 1 gene every 4600bp = 217 genes/Mb

Drosophila: 1 gene every 9000bp = 111 genes/Mb

6. Page 15, lines 409-411, “S. microadriaticum has a relatively low number of repetitive elements, comprising only 26.5% of the genome compared to 37.5% in mouse, ~50% in human, and 84.7% in wheat”. As above, perhaps a comparison with yeast, C. elegans and Drosophila would be more appropriate. Do the LINEs encode a reverse transcriptase and do they have a similar organization to LINEs in humans or are they called LINEs because they are long and they don’t have LTRs?

Response: we have now examined repeat densities in yeast, C. elegans and Drosophila. We find that repeat density is comparable to that in Drosophila. Due to word limits we no longer discuss how repeat density compares to other species.

7. Page 21, lines 520-521, “a strong reduction is observed at convergent boundaries”. Whether the observed reduction is strong or minor is subjective. Please replace “strong” and “minor” for the actual numbers and let the reader decide.

Response: the reviewer is correct. We have reworded this sentence given the very small GC change at divergent sites. In the revised manuscript we state:

“Finally, a clear reduction in GC content (~10%) is only observed at convergent boundaries (Figure 5F, 5H)”

8. Discussion, lines 702-705. “We performed Hi-C using a combination of formaldehyde and DSG. The Hi-C interaction maps and P(s) plots obtained this way are very similar (Supplemental Figure S8), suggesting the small exponent is not due to low cross-linking efficiency”. Move to results?

Response: Due to space limitations we prefer to keep this result in the Extended Data Figures, so that we can dedicate the main text to the key findings.

Reviewer #3:
Remarks to the Author:
By combining Hi-C and PacBio sequencing, Nand, Zhan and colleagues assemble and provide the first version reference genome for the dinoflagellate Symbiodinium microadriaticum. Nowadays, de novo genome assembling is not technically challenging and even not particular interesting to some biologists. However, providing a reference genome for a species not previously sequenced is of great value to the scientific community, not to mention in this case, a very unique and important organism.

The authors then analyze the genomic sequence including GC content, gene density, gene orientation, repetitive elements and so on; routine and basic analysis. Many unique features are revealed. Gene density increases towards the ends of chromosomes. Genes are arranged in unidirectional blocks alternating between top and bottom strands. Genes involved in specific biological processes are enriched in some chromosomes. These genetic features are quite different from eukaryotic model organisms that are mostly used in laboratories.

Besides, the genome of S. microadriaticum is found being organized into structural domains, similar to topologically associating domains (TADs), and these domains are separated at sites where transcription of two gene blocks converge, suggesting a strong correlation between the linear genetic sequence and three-dimensional organization.

Dinoflagellates chromosomes exist in a unique state and are not packaged by histones, which is dramatically different from metazoans analyzed so far. Chromosomes in a special state provide a unique window for better understanding elusive genome folding mechanisms and function-structure relationship, for example, plant vs animal, inactive X chromosome vs autosomes, cells in M phase vs interphase, sperm vs somatic cells, and many other comparisons.

To me, this is a quite impressive work on a species that is not usually used for genetic study. Nevertheless, there are a few concerns that should be further clarified by the authors.

Response: We thank the reviewer for their supportive comments on our manuscript.

Major comments:

1. The reference genome assembling is a very important part of this work. However, a total of 183,768,579 bp were set aside, more than one fifth of the whole genome. These excluded sequences interact frequently with more than one chromosome (line 164-166). After clustering these sequences into 94 groups, the authors wrote “each of these clusters interacts particularly frequently with only one of the 94 chromosomes, and each chromosome interacts frequently

with sub-scaffolds from only one such cluster” (Line 168-170). It seems to me that a specific sub-scaffold, if clustered in one specific cluster, then it is supposed to interact frequently with a specific chromosome. Since these clustered sub-scaffolds interact frequently with only one of the 94 chromosomes (Figure 1C), the authors may want to think about trying other technologies to see if these large amounts of sequences can be placed at proper locations in the genome assembly. This will surely improve the completeness of
this reference genome, and this should be doable.

Response: We thank the reviewer for encouraging us to try another round of improving the assembly. We generated new PacBio long-read based contigs and used these for extensive gap filling to produce a new assembly: Smic1.1N. In this new assembly we have added >110 Mb of DNA, **and were able to place on assembled chromosomes more than 50% of the ~180 Mb we could not place before**. We believe this addresses a major concern raised by this reviewer. We note that by incorporating new sequences that are often highly repetitive (as we had shown in the first version of the manuscript), we also introduced new complications (illustrated in the updated Extended Data Fig. 2). In the revised manuscript we describe the original assembly Smic1.0 that is extensively manually curated and is conservative but reliable scaffold with gaps, as well as the newly gap-filled assembly Smic1.1N that contains more sequences but is not manually curated. We perform all analyses on both assemblies with almost identical results. All results we show for Smic1.0 in the main figures are shown for Smic1.1N in the Extended Data Figures. We have made both assemblies and gene annotation for assemblies available through GEO.

2. The authors find that the genome of S. microadriaticum is partitioned into domain structures. In mammalian cells, chromosome compartments and TADs are lost in metaphase, and chromosome structure described for nonsynchronous cells is restricted to interphase (Naumova et. al., Science, 2013). In this paper, Hi-C maps of G2/M immobile and G1/S flagellated cells are compared, and no obvious differences revealed (Supplemental Figure S2, line 193-195). The authors should explain if cells of this specific species in G2 phase have the same very condensed chromosomes as in M phase and different from mammalian G2 phase, or vice versa. Or if through the whole cell cycle, the chromosomes are consistently in a non-changing state. Whichever it is, the authors may want to provide microscopic images to show if and how the genome/chromosomes changes through the cell cycle fluctuating between condensed and relaxed states as in mammalian cells. Otherwise, the comparison between G2/M and G1/S phase and the claim that the domain structure does not change seem not valid to me.

Response: The reviewer brings up a very good point. Dinoflagellate chromosomes have been extensively studied during the cell cycle using microscopic observations, and their permanent condensed state is well established. A key publication is Bhaud et al. (2000) "Morphology and behaviour of dinoflagellate chromosomes during the cell cycle and mitosis" J. Cell Science 113, 1231-1239. This study showed some minor changes in chromosome condensation during the cell cycle but chromosomes remain condensed as rods. We have imaged chromosomes in G2/M immobile and G1/S flagellated cells and in both types of cells we observe condensed rod-shaped chromosomes. If the reviewer and editor feels this is critical to add we can add images of these chromosomes as an Extended Data Figure panel. However, the cell cycle synchronization is not very good in these cultures. We spent significant effort to arrest cells in various cell cycle stages but we have not been able to establish experimental conditions for cell cycle synchronization of *S. microadriaticum* cultures

Minor comments:

1. The exclusion of sub-scaffolds does not affect the 3D genome architecture analysis (line 214-217), however, we cannot rule out the possibility that their distribution pattern in the genome may affect the GC content, gene density analysis, and also other analyses.

Response: We have now been able to include > 90 Mb of the repetitive sub-scaffold in the assembled chromosomes in the new assembly Smic1.1N. We have repeated all analyses, including GC content, gene density, domains formation etc. All results are robustly reproduced even after inserting at least half of these repetitive sub-scaffolds. These analyses are shown in the new/updated Extended Data Figures 2, 4, 5, 7 and 8.

2. Compartmentalization of chromosomes is another structural feature of many eukaryotic genomes. It seems to me that compartment-like patterns can also been identified in the Hi-C heatmaps (Figure 1C). The authors may want to further analyze if domain structures preferentially interact within compartments as well in this species.

Response: We agree that compartmentalization could be further analyzed. Compartmentalization is typically detected in Hi-C by the appearance of a checkerboard pattern. However, we do not recognize any checkerboard patterns in the Hi-C maps. For instance if highly transcriptionally active regions preferentially interact (as in the A-compartment in many other eukaryotes) we would expect to see enriched interactions between the two telomeric ends of the chromosomes. No such enrichment is observed. Therefore, we do not know how to analyze compartmentalization further at this point.

3. It is quite interesting that the domain borders are frequently found at the convergent sites of two unidirectional gene blocks. This reminds me of a work published showing that genes flanking borders were also found convergent in the Drosophila genome (Hou et. al., Mol Cell, 2012). Genes in the genome of Drosophia are not so neatly organized into unidirectional blocks as in S. microadriaticum. If this is just a coincidence or it implies a conserved mechanism can be discussed.

Response: The reviewer brings up an excellent point: there are various examples in the literature where gene expression and position are related to chromosomal domain formation. We mention several, including bacteria. In the revised manuscript we now also include Drosophila.

4. It would be better if mechanistic studies can be carried out to test if transcription is playing a role in the domain structure establishment. Given the extensive results reported in this article, this may not be a prerequisite for its publication.

Response: In the revised manuscript we now show experiments where we attempted to block transcription and describe that such treatment leads to loss of domains. Specifically, we have treated cells with two chemicals known to block transcription in other eukaryotes. This treatment led to growth arrest. Hi-C data show that this treatment also led to significant changes in chromosome folding, with domain boundaries disappearing. These new exciting data are shown in the newly added Figure 6. We were not able to identify conditions where S*. microadriaticum* takes up modified bases such as EU, and therefore we were not able to ascertain that this treatment indeed blocks nascent transcription. However, based on the data shown here we can conclude that chromosome conformation can be modulated under conditions that block cell growth, *possibly* through effects on transcription. Importantly, the fact that domain formation is sensitive to growth conditions shows domain boundaries are not due to remaining genome assembly errors.

Overall, this work is quite interesting to me. The results provide not only a valuable reference genome but also many discoveries of unique genetic and structural features in the genome of a special species. It may very likely open a window for people to better understand how the genome is organized from an evolutionary, and/or function-structure related point of view.

Response: Thank you for this supporting statement. We agree and hope that this work is of interest to a wide community of chromosome biologists.

Decision Letter, first revision:

26th Jan 2021

Dear Job,

Thank you for submitting your revised manuscript "Genetic and spatial organization of the peculiar chromosomes of the dinoflagellate Symbiodinium microadriaticum" (NG-A55056R). It has now been seen by the original referees and their comments are below. The reviewers find that the paper has improved in revision, and therefore we will be happy in principle to publish it in Nature Genetics, pending minor revisions to satisfy the referees' final requests and to comply with our editorial and formatting guidelines.

** Note that we will send you a checklist detailing these editorial and formatting requirements in about a week. Please do not finalize your revisions or upload the final materials until you receive this additional information.**

In recognition of the time and expertise our reviewers provide to Nature Genetics's editorial process, we would like to formally acknowledge their contribution to the external peer review of your manuscript entitled "Genetic and spatial organization of the peculiar chromosomes of the dinoflagellate Symbiodinium microadriaticum". For those reviewers who give their assent, we will be publishing their names alongside the published article.

While we prepare these instructions, we encourage the Corresponding Author to begin to review and collect the following:

-- Confirmation from all authors that the manuscript correctly states their names, institutional affiliations, funding IDs, consortium membership and roles, author or collaborator status, and author contributions.

-- Declarations of any financial and non-financial competing interests from any author. For the sake of transparency and to help readers form their own judgment of potential bias, the Nature Research Journals require authors to declare any financial and non-financial competing interests in relation to the work described in the submitted manuscript. This declaration must be complete, including author initials, in the final manuscript text.

If you have any questions as you begin to prepare your submission please feel free to contact our Editorial offices at genetics@us.nature.com. We are happy to assist you.

Thank you again for your interest in Nature Genetics and congratulations!

Sincerely,

natureresearch

Tiago

Tiago Faial, PhD
Senior Editor
Nature Genetics
https://orcid.org/0000-0003-0864-1200

Reviewer #1:

I think the authors have responded to my comments sufficiently. Thanks.

Reviewer #2:

The authors have addressed all my original concerns. This is a very nice piece of work that will be of interest to a large audience of biologist. The experiments are rigorously performed and analyzed, conclusions are carefully interpreted, and the logic of the manuscript flows very well after the re-arrangement of the various sections. I like that the authors didn't make up a confusing name for the domains they see. I only have a few minor suggestions/typos below

1. In line 51, authors may consider eliminating the parentheses flanking “a portion of”.

2. Line 59. Spell out DVNPs

3. Line 306. “the formation of relatively stiff….”?

4. Lines 415-416. “as cultures resumed growth with a normal doubling rate after the chemicals were washed away”

Reviewer #3:

The authors have carefully revised their manuscript and addressed my concerns adequately. I am satisfied with this revised version and have no further comments.

ORCID

Nature Genetics is committed to improving transparency in authorship. As part of our efforts in this direction, we are now requesting that all authors identified as 'corresponding author' create and link their Open Researcher and Contributor Identifier (ORCID) with their account on the Manuscript Tracking System (MTS) prior to acceptance. ORCID helps the scientific community achieve unambiguous attribution of all scholarly contributions. For more information please visit http://www.springernature.com/orcid

For all corresponding authors listed on the manuscript, please follow the instructions in the link below to link your ORCID to your account on our MTS before submitting the final version of the manuscript. If you do not yet have an ORCID you will be able to create one in minutes. https://www.springernature.com/gp/researchers/orcid/orcid-for-nature-research

IMPORTANT: All authors identified as 'corresponding author' on the manuscript must follow these instructions. Non-corresponding authors do not have to link their ORCIDs but are encouraged to do so. Please note that it will not be possible to add/modify ORCIDs at proof. Thus, if they wish to have their ORCID added to the paper they must also follow the above procedure prior to acceptance.

To support ORCID's aims, we only allow a single ORCID identifier to be attached to one account. If you have any issues attaching an ORCID identifier to your MTS account, please contact the Platform Support Helpdesk.

Author Rebuttal, first revision:

Response to reviewers

We thank all three reviewers for their supportive comments.

Reviewer #2:
Remarks to the Author:
The authors have addressed all my original concerns. This is a very nice piece of work that will be of interest to a large audience of biologist. The experiments are rigorously performed and analyzed, conclusions are carefully interpreted, and the logic of the manuscript flows very well after the re-arrangement of the various sections. I like that the authors didn't make up a confusing name for the domains they see. I only have a few minor suggestions/typos below

1. In line 51, authors may consider eliminating the parentheses flanking "a portion of".

2. Line 59. Spell out DVNPs

3. Line 306. "the formation of relatively stiff…."?

4. Lines 415-416. “as cultures resumed growth with a normal doubling rate after the chemicals were washed away”

Response: We thank the reviewer and have corrected these typos in the revised manuscript.

Final Decision Letter:

9th Mar 2021

Dear Job,

I am delighted to say that your manuscript "Genetic and spatial organization of the unusual chromosomes of the dinoflagellate Symbiodinium microadriaticum" has been accepted for publication in an upcoming issue of Nature Genetics.

Prior to setting your manuscript, we may make minor changes to enhance the lucidity of the text and with reference to our house style. We therefore ask that you examine the proofs most carefully to ensure that we have not inadvertently altered the sense of your text in any way.

Once your manuscript is typeset and you have completed the appropriate grant of rights, you will receive a link to your electronic proof via email with a request to make any corrections within 48 hours. If, when you receive your proof, you cannot meet this deadline, please inform us at rjsproduction@springernature.com immediately.

Your paper will be published online after we receive your corrections and will appear in print in the next available issue. You can find out your date of online publication by contacting the Nature Press Office (press@nature.com) after sending your e-proof corrections. Now is the time to inform your Public Relations or Press Office about your paper, as they might be interested in promoting its publication. This will allow them time to prepare an accurate and satisfactory press release. Include your manuscript tracking number (NG-A55056R1) and the name of the journal, which they will need when they contact our Press Office.

Before your paper is published online, we shall be distributing a press release to news organizations worldwide, which may very well include details of your work. We are happy for your institution or funding agency to prepare its own press release, but it must mention the embargo date and Nature Genetics. Our Press Office may contact you closer to the time of publication, but if you or your Press Office have any enquiries in the meantime, please contact press@nature.com.

Acceptance is conditional on the data in the manuscript not being published elsewhere, or announced in the print or electronic media, until the embargo/publication date. These restrictions are not intended to deter you from presenting your data at academic meetings and conferences, but any enquiries from the media about papers not yet scheduled for publication should be referred to us.

Nature Genetics is a Transformative open access journal. In approximately 10 business days you will receive an email with a link to choose the appropriate publishing options for your paper and our

naturerESEARCH

Author Services team will be in touch regarding any additional information that may be required.

Please note that Nature Research offers an immediate open access option only for papers that were first submitted after 1 January, 2021.

You will not receive your proofs until the publishing agreement has been received through our system.

If you have any questions about our publishing options, costs, Open Access requirements, or our legal forms, please contact ASJournals@springernature.com

In the event that you choose to publish under the subscription model, Nature Research allows authors to self-archive the accepted manuscript (the version post-peer review, but prior to copy-editing and typesetting) on their own personal website and/or in an institutional or funder repository where it can be made publicly accessible 6 months after first publication, in accordance with our self-archiving policy. Please review our self-archving policy for more information.

Several funders require deposition the accepted manuscript (AM) to PubMed Central or Europe PubMed Central. To enable compliance with these requirements, Nature Research therefore offers a free manuscript deposition service for original research papers supported by a number of PMC/EPMC participating funders. If you do not choose to publish immediate open access, we can deposit the accepted manuscript in PMC/Europe PMC on your behalf, if you authorise us to do so.

If you have posted a preprint on any preprint server, please ensure that the preprint details are updated with a publication reference, including the DOI and a URL to the published version of the article on the journal website.

To assist our authors in disseminating their research to the broader community, our SharedIt initiative provides you with a unique shareable link that will allow anyone (with or without a subscription) to read the published article. Recipients of the link with a subscription will also be able to download and print the PDF.

As soon as your article is published, you will receive an automated email with your shareable link.

You can now use a single sign-on for all your accounts, view the status of all your manuscript submissions and reviews, access usage statistics for your published articles and download a record of your refereeing activity for the Nature journals.

An online order form for reprints of your paper is available at https://www.nature.com/reprints/author-reprints.html. Please let your coauthors and your institutions' public affairs office know that they are also welcome to order reprints by this method.

Sincerely,

Tiago

Tiago Faial, PhD
Senior Editor
Nature Genetics
https://orcid.org/0000-0003-0864-1200

Click here if you would like to recommend Nature Genetics to your librarian
http://www.nature.com/subscriptions/recommend.html#forms

** Visit the Springer Nature Editorial and Publishing website at www.springernature.com/editorial-and-publishing-jobs for more information about our career opportunities. If you have any questions please click here.**
